# CpG islands under selective pressure are enriched with H3K4me3, H3K27ac and H3K36me3 histone modifications

**DOI:** 10.1186/1471-2148-13-145

**Published:** 2013-07-10

**Authors:** Most Mauluda Akhtar, Giovanni Scala, Sergio Cocozza, Gennaro Miele, Antonella Monticelli

**Affiliations:** 1 “Gruppo Interdipartimentale di Bioinformatica e Biologia Computazionale, Università di Napoli “Federico II” - Università di Salerno, Naples, Italy; 2 Dipartimento di Fisica, Università degli Studi di Napoli “Federico II”, Naples, Italy; 3 Istituto Nazionale di Fisica Nucleare – Sezione di Napoli, Naples, Italy; 4 Dipartimento di Medicina Molecolare e Biotecnologie Mediche, Università degli Studi di Napoli “Federico II”, Naples, Italy; 5 CNR, Istituto di Endocrinologia ed Oncologia Sperimentale IEOS, Naples, Italy

**Keywords:** Epigenetic, Selective pressure, CpG islands, H3K4me3, H3K27ac, H3K36me3

## Abstract

**Background:**

Histone modification is an epigenetic mechanism that influences gene regulation in eukaryotes. In particular, histone modifications in CpG islands (CGIs) are associated with different chromatin states and with transcription activity. Changes in gene expression play a crucial role in adaptation and evolution.

**Results:**

In this paper, we have studied, using a computational biology approach, the relationship between histone modifications in CGIs and selective pressure in *Homo sapiens*. We considered three histone modifications: histone H3 lysine 4 trimethylation (H3K4me3), histone H3 lysine 27 acetylation (H3K27ac) and histone H3 lysine 36 trimethylation (H3K36me3), and we used the publicly available genomic-scale histone modification data of thirteen human cell lines. To define regions under selective pressure, we used three distinct signatures that mark selective events from different evolutionary periods. We found that CGIs under selective pressure showed significant enrichments for histone modifications.

**Conclusion:**

Our result suggests that, CGIs that have undergone selective events are characterized by epigenetic signatures, in particular, histone modifications that are distinct from CGIs with no evidence of selection.

## Background

CpG islands (CGIs) are unmethylated segments of a genome that have an increased level of CpG dinucleotides and a high GC content [1,2]. In the human genome, most CGIs are either inside or close to the promoter regions of genes[3]. Typically these CGIs occur at or close to transcription start sites (TSSs) [4]. It is well established that CpG sites in promoter CGIs are undermethylated in expressed genes, while hypermethylation of promoter CpG sites is associated with gene silencing [5]. Others CGIs that are distant from known TSSs have been found in intergenic, 3’ and intragenic regions [6].

There is an extensive literature demonstrating that structural modifications to chromatin, along with CGI methylation, contribute to the functional output of related genes [7]. The N-terminal tails of histone proteins can be modified covalently by small molecules (for example, phosphorylation, acetylation, methylation) and by macromolecules (for example, ubiquitination, sumoylation etc.). The precise environment of the CGI chromatin that controls gene regulation is not definitively established. The general understanding is that by altering the state of the CGI chromatin, histone modification can regulate access of the transcription machinery to particular DNA sequences [8]. Of all the possible histone modifications, methylation of the lysine or arginine residues has received the main attention. These modifications can activate or repress the associated genes depending on which lysine or arginine residues are methylated [9]. Methylation of histone H3 at lysine 9 (H3K9) or lysine 27 (H3K27) is considered to be a repressive mark [9]. In contrast, H3K4me3, perhaps the best established epigenetic marker, is robustly associated with activation of transcription [9]. In mammals, the trimethylation of H3K4 can be catalyzed by different histone methyltransferases, such as MLL1 or ASH1L [10,11]. The majority of H3K4me3 sites overlap with the 5’ ends of annotated human genes [12] and several studies have reported the inverse correlation between two epigenetic marks, DNA methylation and H3K4me3 [13,14]. The H3K4me3 mark also plays a crucial role in mammalian development [15], and its alteration has been found to be associated with cancer and other diseases [16-18]. In addition, both H3K27ac and H3K36me3, which are known as a promoter mark [19] and a gene body mark [20], respectively, are associated with transcriptional activation [19,21].

Alterations in gene regulation are thought to play an important role both in adaptation and evolution [22]. A recent report proposed that differences in gene expression levels among primates are associated with the changes in H3K4me3 [23]. Moreover, another recent study identified human-specific changes in H3K4me3 levels at TSSs and related regulatory sequences in comparison with chimpanzees and macaques [24]. Besides, the Encyclopedia of DNA Elements (ENCODE) project is studying different functional elements of human genome including regions of histone modifications. In particular they assayed chromosomal locations for 12 histone modification in 46 different cell types [25]. In a previous study [26], we demonstrated that CGIs under selective pressure are hypomethylated compared to the CGIs in other regions of the genome. In this study, we explored the relationship between selective pressure signatures of and histone modification (H3K4me3, H3K27ac and H3K36me3) enrichment in CGIs. We used the genome-wide histone modification data of thirteen human cell lines produced by the ENCODE consortium [27,28]. To define regions under selective pressure we used three distinct methods [26] that are able to detect both recent and ancient selective pressure events [29].

## Results

We analyzed thirteen cell lines from the ENCODE/Broad Institute, derived from nine normal and four cancer tissues, respectively. A list of features for each considered cell line is presented in Additional file 1: Table S1. For each cell line, we downloaded histone modification data for H3K4me3, H3K27ac and H3K36me3 marks.

We used the “Peaks Signal” (PS), representing regions of statically significant enrichment of a specific histone modification (see Materials and Methods). We downloaded genomic coordinates of 27718 unique CGIs defined according to criteria described in the University of California Santa Cruz Genome Browser (UCSC GB) ( http://genome.ucsc.edu/) (see Materials and Methods). For each cell line, we estimated the number of CGIs containing at least one PS of histone modification and found, on average, 15478, 11903 and 10182 CGIs containing PSs of H3K4me3, H3K27ac and H3K36me3, respectively.

To identify genomic regions that may have undergone selective pressure we used three different approaches that are sensitive to selective pressure events that occurred in distinct evolutionary epochs.

The first method uses the per-continent “integrated Haplotype Score” (iHS) [30] and marks recent positive selection (see Materials and Methods). Using the iHS we identified 586 genomic regions that have putatively undergone recent selective pressure. We denoted these regions as “high iHS regions” (HIRs). Within the HIRs regions we found 2545 CGIs.

The second approach is based on a comparison between *Homo sapiens* and *Neanderthal* genomes (see Materials and Methods). The selective sweep scan score (S score) was used to identify regions of the human genome with a strong signal for depletion of Neanderthal-derived alleles. This score, when negative, may indicate an episode of positive selection in early humans [31]. We found 212 genomic regions with a significant negative score (5% lowest S regions, hereafter denoted as 5LSRs) containing 348 CGIs.

In the third approach, we looked for sequences that were conserved across ten primate genomes. These sequences are the so-called “Conserved Elements” (CEs) (see Materials and Methods) and they mark ancient selective pressure events. We downloaded 725627 CEs and used them to search for CGIs that contain CEs [32]. We identified 13288 unique CGIs that contained at least one CE.

We, then, computed the fraction of CGIs containing histone modification marks that show signatures of natural selection (HIRs, CEs and 5LSRs), and compared it with an analogous quantity computed for CGIs shown to have no signals of selective pressure. The presence of a possible enrichment/diminishment, defined as the ratio of the percentages of the above two groups, was assessed by means of a hypergeometric test (see Material and Methods).

### Overall Analysis

**Figure 1:**
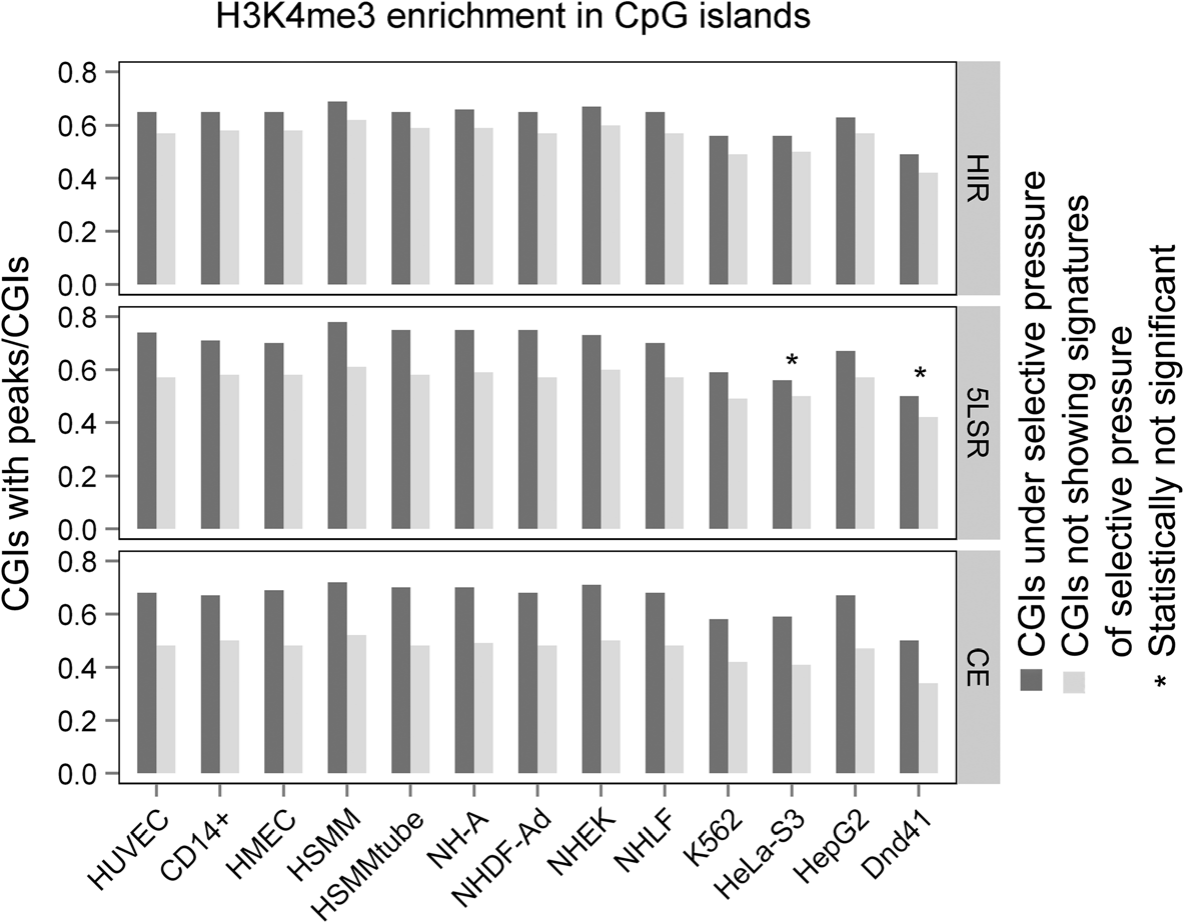
**Enrichment of H3K4me3 modification in CpG islands under selective pressure.** Black bars represent the fraction of CGIs containing histone modification marks within regions that show signatures of natural selection (HIRs, CEs and 5LSRs). Grey bars represent the fraction of CGIs containing histone modification marks within regions that do not show signatures of selective events. The X-axis indicates the analyzed cell lines. An asterisk (*) above a bar indicates a statistically non-significant difference.

**Figure 2:**
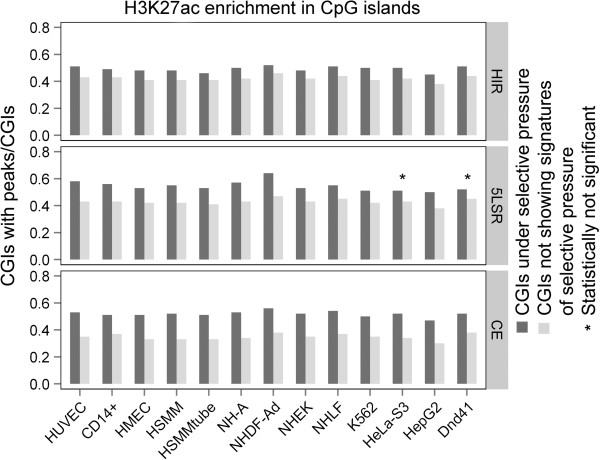
**Enrichment of H3K27ac modification in CpG islands under selective pressure.** Same notation as in Figure 1.

**Figure 3:**
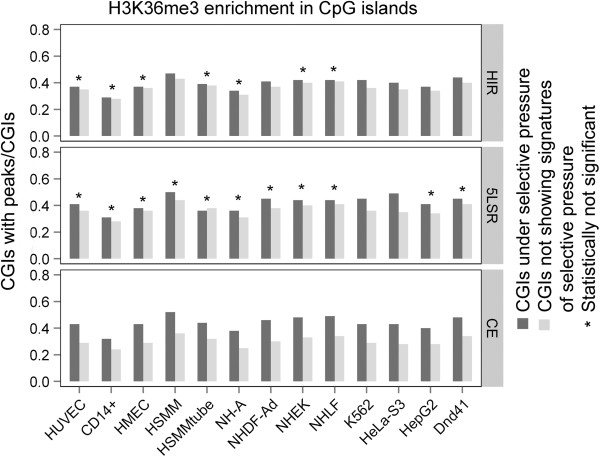
**Enrichment of H3K36me3 modification in CpG islands under selective pressure.** Same notation as in Figure 1.

We found a significant enrichment of H3K4me3 and H3K27ac markers for all three signatures of selection in almost all cell lines (Figure 1, Figure 2 and Additional file 2: Table S2) while for H3K36me3 the enrichment reached significance only for the CE signature (Figure 3 and Additional file 2: Table S2).

In order to understand if the results are due to the same CGIs identified by the three different methods, we estimated the overlaps among the corresponding CGIs lists (see Additional file 3). As shown in the figure, the different sets of CGIs identified are not essentially included one in the other.

### Position analysis

We investigated whether or not these differences were dependent on the position of the CGIs in various genomic regions. To do this we followed the same approach described by Medvedeva et al. [6] dividing the CGIs into four groups according to their positions with respect to genes: at the 5’ end of a gene, in the intragenic region, at the 3’ end of a gene, and in the intergenic region. Results of this analysis are presented as additional information (see Additional file 4: Table S3) and summarized below.

Analysis of 5’ CGIs demonstrated the same significant enrichment pattern as seen in the overall analysis with significant enrichment of H3K4me3 and H3K27ac (Additional file 5 and Additional file 6), in almost all cell lines for all signatures of selection, and significant enrichment of H3K36me3 in all cell lines for the CE signature only (Additional file 7).

Both intragenic and 3’ CGIs were significantly enriched for H3K36me3 in the majority of cells lines (nine and ten out of the thirteen, respectively) for the CE signature (Additional file 8 and Additional file 9), while analysis of the other markers did not reach significance in almost all other cases (Additional file 10, Additional file 11, Additional file 12 and Additional file 13).

Finally, regarding intergenic CGIs we found a significant enrichment in all cell lines for all considered markers for the CE signature and in twelve out of thirteen cell lines for H3K4me3 in the HIR signature (Additional file 14, Additional file 15 and Additional file 16).

### Evolutionary forces analysis

Two major evolutionary forces result in enriched CpG content: one is based on low levels of DNA methylation and, consequentially, deamination; and the other is biased gene conversion (BGC), which acts to repair TG mismatches caused by the deamination of methyl-cytosine [33]. According to the role that these two forces play in CGI maintenance, CGIs can be classified as hypo-deaminated CGIs or BGC CGIs. We examined whether or not the relationship that we found between selective pressure and histone mark enrichment was present in both classes.

We found that both hypo-deaminated and BCG CGIs showed an enrichment of all markers in the CE signature in all cell lines, while only BGG CGIs showed significant enrichment of H3K4me3 and H3K27ac in the HIR signature in all cell lines (Additional file 17, Additional file 18, Additional file 19, Additional file 20, Additional file 21, Additional file 22 and Additional file 23: Table S4).

### Expression divergence between humans and chimps and histone modifications

Cain et al. [23] have identified genes whose expression levels differ between species. In particular, by using their approach we have classified 1888 genes as Differentially Expressed (DE) between humans and chimpanzees, at a FDR of 0.050 (see Materials and Methods) among the whole set of 12559 genes considered in their study [23]. Inside this class, and by applying a GREAT analysis [34] we associated corresponding sets of genes to CE CGIs (7436), HIR CGIs (1190) and 5SLR CGIs (214) (see Materials and Methods). As shown in Additional file 24: Table S5, the CE CGI genes are significantly enriched for DE, while HIR CGI genes, even if they do not match our conservative threshold for statistical significance of 0.001, show a p-value = 0.008. No significant signal is present in 5SLR CGI genes. In the Additional file 25: Table S6 we presented the histone modification enrichments referred to the different classes of genes labeled by their selective signal (present or not), and by their possible membership to the class of DE. As clear from the table, we found a significant enrichment of histone modifications in DE + CE genes, with respect to DE – CE class (defined as the set of elements in DE not belonging to CE), and this is independent of the histone modification chosen. The same is not true when we compare the enrichment in DE + CE genes, with respect to CE – DE. This suggests that CE plays the central role for the enrichment. The same is true for HIR if we only consider H3K4me3, whereas we get at most a marginal statistical significance if we take H3K27ac and H3K36me3. No significance at all is found for 5SLR.

As discussed in Materials and Methods, we assumed the conservative approach to define as PS genes the ones associated to the list of PS CGIs that were simultaneously present in all cell lines. In order to understand if our results depend on such choice we have also considered the other extreme case, namely labeling as PS genes the ones associated to a PS CGIs in at least one cell line. This approach confirms the previous finding (data not shown).

## Discussion

In this study, we investigated the hypothesis that CGIs under selective pressure are enriched with histone modifications that are associated with gene activation. To do this, we analyzed data from thirteen human cell lines for three well-known histone modifications (H3K4me3, H3K27ac and H3K36me3) to explore their relationship with both recent and ancient events of selective pressure.

H3K4me3 and H3K27ac are epigenetic marks that are generally associated with gene activation [9,19] while H3K36me3 is associated with transcriptional elongation [8]. Moreover, H3K4me3 and H3K27ac are evolutionarily conserved among species [35] and negatively correlated with DNA methylation [13,14,36,37]. Also H3K36me3 in exons is found to be conserved between human and mouse [38].

Using the entire set of human CGIs we found that the CGIs associated with signatures of selective pressure were significantly enriched with H3K4me3 and H3K27ac in almost all considered cell lines. H3K36me3, on the other hand, showed a significant enrichment in global CGIs only in CE regions: this could be due to a small sample size effect (Additional file 2). These findings support a previous study in which we found that CGIs located in regions under selective pressure are more protected from DNA methylation compared the CGIs in other genomic regions [26]. In the same study, we found that CGIs under selective pressure show a lower SNP content as well. Here we checked two parameters more: C+G content and CGIs’ length, and we found that CE CGIs have a slight but statistically higher G+C content compared with the remaining CGIs (mean = 0.689 vs. 0.683, t-test p < 2.2 10^-16^), and (adopting the classification described by Elango and Soojin [39]) an enrichment of long (>2000 bp) CGIs (Fisher’s Exact Test p < 2.2 10^-16^). We did not find significant differences in length and G+C content for HIR and 5LSR CGIs.

When we divided CGIs according to their positions with respect to the genes, we found that the statistical differences between CGIs with and without signatures of selective pressure were clearest for CGIs located in the 5’ regions for both H3K4me3 and H3K27ac. This result is intriguing in the light of the well established evidence that CGIs at the 5’ ends of genes are involved mainly in the control of gene expression [40]. It is also possible that the small sample size led to a lack of statistical confidence in the results for CGIs in other positions. We noticed a different behavior for H3K36me3. H3K36me3 was the only mark to be enriched in 3’ and intragenic CGIs in CE regions for majority of cell lines; this finding is again intriguing considering that H3K36me3 is reported to be a gene body mark [8,20]. In a recent study, H3K36me3 mark was found to be significantly associated with alternative splicing [41]. It is well known that, alternative splicing is a key reason for protein diversity in higher eukaryotes [42]. It has been a fundamental question in evolutionary study, how species having the similar repertoires of protein-coding genes differ strikingly at the phenotypic level. A very recent study proposed a link between alternative splicing and species-specific phenotypic differences among vertebrate species [43].

Two different evolutionary processes, namely hypo-deamination and BCG, are involved in the generation and maintenance of CGIs [33]. The majority of hypo-deaminated CGIs are usually unmethylated while most BGC CGIs are constitutively methylated and clustered in subtelomeric regions. We found H3K4me3, H3K27ac and H3K36me3 enrichment in CGIs in CE regions, independently of the evolutionary process involved in their generation. Since CGIs belonging to these two groups differ in their DNA methylation levels, our finding seems to suggest that the difference we found was quite independent of the DNA methylation status.

The impact of natural selection on functional elements in human genome is also addressed in the last report from ENCODE project [25]. In that case the authors focused their attention mainly on the relationship between negative selection and a subset of functional elements but they did not specifically address histone modifications. Positive selection, on the other hand, was addressed in a recent work by Vernot et al. [44] who studied the impact of this kind of selective pressure on DNase I peaks.

Cain et al [23] identified a list of genes that were differentially expressed between humans and other primates. They proposed that epigenetic changes could be, at least in part, involved in these differences. When we compared this list with the list of genes associated to the CGIs, we found a significant enrichment of differentially expressed genes in the CE CGI genes list, while the HIR CGI genes showed a p-value (0.008) near our conservative threshold for statistical significance (0.001). This seems to suggest that genes transcriptionally different between species are more likely located near CGIs with signature of selective pressure. In literature it is growing the evidence that epigenetic mechanisms provide a significant source of phenotypic variation that, in turn, can cause evolutionary novelty and potentially influence adaptation and evolution. Although the exact evolutionary significance of our results need further experiments to be completely defined, our data seem to support the idea of a close connection between adaptation, evolution and epigenetic mechanisms.

It has been hypothesized that CGIs are fundamental regulatory structures that have evolved under selection in genomes where DNA methylation plays a regulatory role [24,45]. In particular, CGIs act as a platform where chromatin modifications and additional signaling help to define the functional output of the respective genes.

The present analysis concerning H3K4me3, H3K27ac and H3K36me3 enrichment in CGIs under selective pressure, supplements the findings of a previous paper by Cocozza et al. [26]. In that study, we demonstrated a DNA undermethylation of CGIs under selective pressure. It is well established that a complex, perhaps bidirectional, crosstalk exists between DNA methylation and histone modification [45] suggesting that these two epigenetic mechanisms are, at some extent, dependent one each other. The overall picture emerging from the two studies is that CGIs under selective pressure seem to share definite epigenetic features.

To our knowledge, the present study is the first report addressing the relationship between histone modifications and natural selection and the overall framework emerging from our analyses support the hypothesis that CGIs that have experienced selection could be characterized by distinct epigenetic signatures.

## Conclusion

Analyzing thirteen human cell lines, we found H3K4me3, H3K27ac and H3K36me3 enrichment in the CGIs that experienced selective events. Further studies using other epigenetic marks could help to clarify the relation between epigenetic modification and selective pressure in human genome.

## Methods

### UCSC CGIs

CGIs coordinates were downloaded from the “CpgIslandExt” track of the UCSC GB ( http://genome.ucsc.edu/). The CGIs in this track were predicted by searching the human genome assembly (GRCh37/hg19) sequence, scoring each dinucleotide and identifying maximally scoring segments. In this dataset, a CpG island was defined according to the following criteria: i) GC content of 50% or greater, ii) length of at least 200 bp, and iii) observed CpG / expected CpG ratio greater than 0.6. The CGI set that we obtained consisted of 27718 CGIs (this excluded the CGIs in the data related to the alternative haplotype sequences).

### 5’, intragenic, 3’, and intergenic CGIs

We used the classification system that was described previously by Medvedeva et al. [6] in which the CGIs were classified according to their locations. Thus, the CGIs were classified into four classes:

1. 5’ CGIs - located in the 5’ flank region (3 kb upstream the TSS), the 5’ UTR-exon, the 5’ UTR-intron, the initial coding exon or the initial intron.

2. Intragenic CGIs are located in the internal exons and introns.

3. 3’ CGIs are located in the final exon, the final introns, the 3’ UTR-exon or in the 3’ UTR-intron.

4. Intergenic CGIs are located at least 3 kb upstream or downstream from any known gene.

### Hypo-deaminated and biased gene conversion (BGC) CGIs

Two sets of CGIs were described by Cohen et al. [33] using a new parameter-rich evolutionary model in combination with high resolution DNA methylation data to study the origin of the CpG repertoire in primate genomes (marmoset, rhesus, orangutan, chimp and human). Following a clustering analysis, they observed that most CGIs were constitutively unmethylated and underwent slow C-to-T deamination. They denoted this group as hypo-deaminated CGIs. In contrast, another class of CGI was constitutively methylated with a rapid deamination rate and was termed as BGC CGIs. For our analysis, we considered the 9091 hypo-deaminated and 4782 BGC CGIs from the UCSC CGIs sample.

### Histone modification data

The histone modification (H3K4me3, H3K27ac and H3K36me3) data for thirteen human cell lines (HUVEC, Monocytes-CD14+_RO01746 (CD14+), HMEC, HSMM, HSMMtube, NH-A, NHDF-Ad, NHEK, NHLF, K562, HeLa-S3, HepG2 and Dnd41) were downloaded from the “Broad histone” track of the UCSC GB. This track contains genome-wide histone modification data of different cell lines, generated using ChIP-seq high-throughput sequencing as a part of the ENCODE project [28]. In this study, we used the “Peaks Signal” (PS), which identifies discrete intervals of ChIP-seq fragment enrichment. In particular, we considered the CGIs in our sample that contained at least one PS.

### Integrated haplotype score (iHS)

The iHS belongs to the Extended Haplotype Homozygosity statistic “family” [46] and is a marker of recent positive selection [30]. The iHS measures the decay of identity, as a function of distance, of haplotypes that carry a specified “core” allele. We downloaded the iHS normalized values from the “HGDP iHS” track of the UCSC GB. The scores were calculated using SNPs genotyped in 1043 individual taken from 53 populations worldwide by the Human Genome Diversity Project in collaboration with the Centre d’Etude du Polymorphisme Humain (HGDP-CEPH). The 53 populations were divided into seven continental groups: Africa (Bantu populations only), Middle East, Europe, South Asia, East Asia, Oceania and the Americas. For each population group, the iHS was calculated and then normalized [30]. Per-SNP iHSs were smoothed in windows of 31 SNPs, centered on each SNP. The final score is -log10 of the proportion of smoothed scores higher than each SNP’s smoothed score. For our analysis, we used the Batch Coordinate Conversion (liftOver) utility (UCSC GB) to convert the genome coordinates from assembly NCBI36/hg18 to assembly GRCh37/hg19. We scanned the normalized iHSs across the whole genome and selected the genomic intervals where the iHS was ≥ 2. After these regions were identified, we extended their boundaries to the nearest loci where the iHS exactly vanished.

### Selective sweep scan (S): the 5% lowest S scores

The S score is based on a comparison between *Homo sapiens* DNA and Neanderthal DNA [31]. We downloaded the regions with S scores from the “5% Lowest S” track of the UCSC GB and denoted them as “5LSRs” (5% lowest S regions). Green et. al. [31] identified polymorphic sites among five modern human genomes and determined the ancestral or derived state of each SNP. The states of the human alleles were used to estimate the expected number of derived alleles in Neanderthal in a 100000-base window around each SNP. The S scores were used to compare the observed number of Neanderthal alleles to the expected number in each window. A positive S score indicates more derived alleles in Neanderthal than expected given the frequency of derived alleles in human; a negative S score, on the other hand, indicates fewer derived alleles in Neanderthal, which might suggest positive selection in the human lineage after divergence from Neanderthal and before divergence in human populations. The 5LSRs represent the regions in the 5% lower percentile of the S score.

### Conserved elements (CEs)

CEs are sequences in the genome that are conserved across species [47]. Conserved regions have a reduced rate of evolution compared to the expected rate under neutral drift. The CEs used in this study were downloaded from the “Conservation (cons46way)” track of the UCSC GB. This track shows measurements of evolutionary conserved elements using two phylogenetic methods, phastCons and phyloP. The CEs used in this study were predicted using ten primates, *Homo sapiens* (reference species), *Pan troglodytes, Gorilla gorilla*, *Pongo pygmaeus abelii*, *Macaca mulatta*, *Papio hamadryas*, *Callithrix jacchus*, *Tarsier syrichta*, *Microcebus murinus* and *Otolemur garnettii*.

### Gene expression data

The complete list of the 12559 genes expressed in lymphoblastoid cell lines LBEG (LymphoBlastoid Expressed Genes) and studied in [23] has been obtained from supplementary data (FileS2.xls) available on “Genetics” journal web site. From this list we selected the subset of 1888 genes differently expressed (DE genes) between *H. sapiens* and *P. troglodytes* using an FDR cut-off of 0.050.

We associated each group of CGIs (CE, HIR, 5SLR) with their putative target genes trough Genomic Regions Enrichment of Annotations Tool (GREAT) by using the default association rule [34] obtaining: 10867 CE CGIs genes, 1726 HIR CGIs genes and 275 5SLR CGIs genes. We then restricted each set of genes to the intersection with LBEG, retaining 7436 CE CGIs genes, 1190 HIR CGIs genes and 214 5SLR genes.

We tested the enrichment of DE in each set of genes associated with the considered signatures of selection by using Fisher’s Exact Test.

To be conservative, we have intersected the list of PS CGIs identified by considering each cell line and each particular histone modification. Hence, the previous GREAT analysis allows labelling as PS the associated genes. We have tested with a Fisher’s Exact Test the enrichment of PS genes inside the classes obtained by overlapping selective signatures and DE.

### Statistical analysis

We used a hypergeometric-based approach to test the null hypothesis that the possible enrichment of H3K4me3, H3K27ac and H3K36me3 is independent of the presence of signals of natural selection. In particular we considered: *k,* the observed number of CGIs containing both PSs and signatures of selective pressure, as the number of success in the sample; *n,* the number of CGIs characterized by signatures of selective pressure only, as the sample size; *M,* the total number of CGIs with PS, as the number of successes in the population; and *N,* the total number of CGIs, as the population size (see Additional file 2: Table S2, Additional file 4: Table S3 and Additional file 23: Table S4). For statistical significance we set the threshold for the Bonferroni corrected p-value at 10^-3^. All the statistical analyses were performed with R ver. 2.14.2 (R Foundation for Statistical Computing, Vienna, Austria; http://www.r-project.org/).

## Abbreviations

CGIs: CpG islands; H3K4me3: Histone H3 lysine 4 trimethylation; H3K27ac: Histone H3 lysine 27 acetylation; H3K36me3: Histone H3 lysine 36 trimethylation; TSS: Transcription start sites; PS: Peaks signal; iHS: Integrated haplotype score; HIR: High iHS regions; S score: Selective sweep scan score; 5LSR: % lowest S regions; CE: Conserved elements; BGC: Biased gene conversion; DE: Differentially expressed.

## Competing interests

The authors declare that they have no competing interests.

## Authors’ contributions

MMA, SC, AM and GM conceived the study and participated in its design and coordination. MMA, GS and SC performed the statistical analysis. MMA, GM, GS and AM helped to draft the manuscript. All authors read and approved the final manuscript.

## Supplementary Material

Additional file 1: Table S1 Characteristics of cell lines used in this study. Click here for file

Additional file 2: Table S2 Raw data used to calculate CGIs enriched with H3K4me3, H3K27ac and H3K36me3 in different cell lines. Click here for file

Additional file 3 Euler diagram showing the overlaps among CGIs localized in the regions under selective pressure detected by the three methods used. Click here for file

Additional file 4: Table S3 Raw data used to calculate CGIs enriched with H3K4me3, H3K27ac and H3K36me3 at 5’, intragenic, 3’ and intergenic locations in different cell lines. Click here for file

Additional file 5 **Enrichment of H3K4me3 modification in 5’ CpG islands under selective pressure.** Black bars represent the fraction of CGIs containing histone modification marks within regions that show signatures of natural selection (HIRs, CEs and 5LSRs). Grey bars represent the fraction of CGIs containing histone modification marks within regions that do not show signatures of selective events. The X-axis indicates the analyzed cell lines. An asterisk (*) above a bar indicates a statistically non-significant difference. Click here for file

Additional file 6 **Enrichment of H3K27ac modification in 5’ CpG islands under selective pressure.** Same notation as Additional file 5. Click here for file

Additional file 7 **Enrichment of H3K36me3 modification in 5’ CpG islands under selective pressure.** Same notation as Additional file 5. Click here for file

Additional file 8 **Enrichment of H3K36me3 modification in intragenic CpG islands under selective pressure.** Same notation as Additional file 5. Click here for file

Additional file 9 **Enrichment of H3K36me3 modification in 3’ CpG islands under selective pressure.** Same notation as Additional file 5. Click here for file

Additional file 10 **Enrichment of H3K4me3 modification in intragenic CpG islands under selective pressure.** Same notation as Additional file 5. Click here for file

Additional file 11 **Enrichment of H3K4me3 modification in 3’ CpG islands under selective pressure.** Same notation as Additional file 5. Click here for file

Additional file 12 **Enrichment of H3K27ac modification in intragenic CpG islands under selective pressure.** Same notation as Additional file 5. Click here for file

Additional file 13 **Enrichment of H3K27ac modification in 3’ CpG islands under selective pressure.** Same notation as Additional file 5. Click here for file

Additional file 14 **Enrichment of H3K4me3 modification in intergenic CpG islands under selective pressure.** Same notation as Additional file 5. Click here for file

Additional file 15 **Enrichment of H3K27ac modification in intergenic CpG islands under selective pressure.** Same notation as Additional file 5. Click here for file

Additional file 16 **Enrichment of H3K36me3 modification in intergenic CpG islands under selective pressure.** Same notation as Additional file 5. Click here for file

Additional file 17 **Enrichment of H3K4me3 modification in hypo-deaminated CpG islands under selective pressure.** Same notation as Additional file 5. Click here for file

Additional file 18 **Enrichment of H3K4me3 modification in BGC CpG islands under selective pressure.** Same notation as Additional file 5. Click here for file

Additional file 19 **Enrichment of H3K27ac modification in hypo-deaminated CpG islands under selective pressure.** Same notation as Additional file 5. Click here for file

Additional file 20 **Enrichment of H3K27ac modification in BGC CpG islands under selective pressure.** Same notation as Additional file 5. Click here for file

Additional file 21 **Enrichment of H3K36me3 modification in hypo-deaminated CpG islands under selective pressure.** Same notation as Additional file 5. Click here for file

Additional file 22 **Enrichment of H3K36me3 modification in BGC CpG islands under selective pressure.** Same notation as Additional file 5. Click here for file

Additional file 23: Table S4 Raw data used to calculate CGIs enriched with H3K4me3, H3K27ac and H3K36me3 in different cell lines according to the CGIs evolutionary model. Click here for file

Additional file 24: Table S5 Lists number of PS genes that are present in each class defined by the possible presence of each signature of selection (“+” stands for presence, “-” stands for absence) and by the membership to DE set (“+” stands for belonging, “-” stands for not belonging). The exact Fisher’s test p-values are reported, highlighting the statistical significant ones. Click here for file

Additional file 25: Table S6 Lists percentages of PS genes for classes defined by the possible presence of each signature of selection (“+” stands for presence, “-” stands for absence) and by the membership to DE set (“+” stands for belonging, “-” stands for not belonging). For each selection signature we give the p-value for the percentages of PS genes in case of DE + compared with DE - (p-values are reported on the same row), and in case of DE + considering the classes of genes under selection or not under selection (p-values are reported on the same column). The p-values are obtained by means of an exact Fisher’s test, highlighting the statistical significant values. Click here for file
