# Supplementary figures and images for: CpG islands under selective pressure are enriched with H3K4me3, H3K27ac and H3K36me3 histone modifications
Source: BMC Evol Biol. 2013 Jul 10;13:145. doi: 10.1186/1471-2148-13-145 (PMC3711888; doi:10.1186/1471-2148-13-145)

**ALL CGI's = 27720**

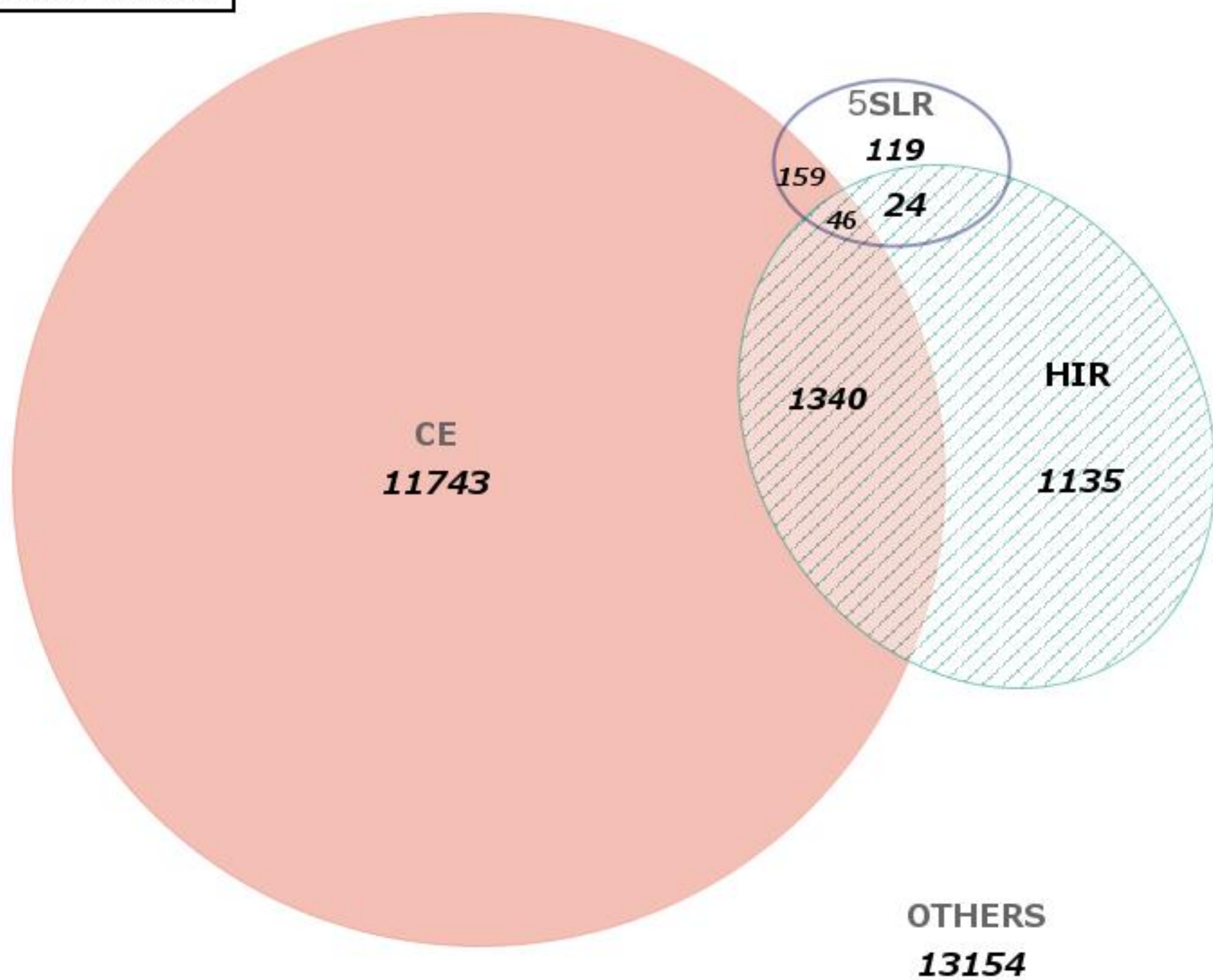

Supplement: Additional file 3 — Euler diagram showing the overlaps among CGIs localized in the regions under selective pressure detected by the three methods used. [file 1471-2148-13-145-S3.pdf]

# H3K27ac enrichment in 5' CGIs

CGIs with peaks/CGIs

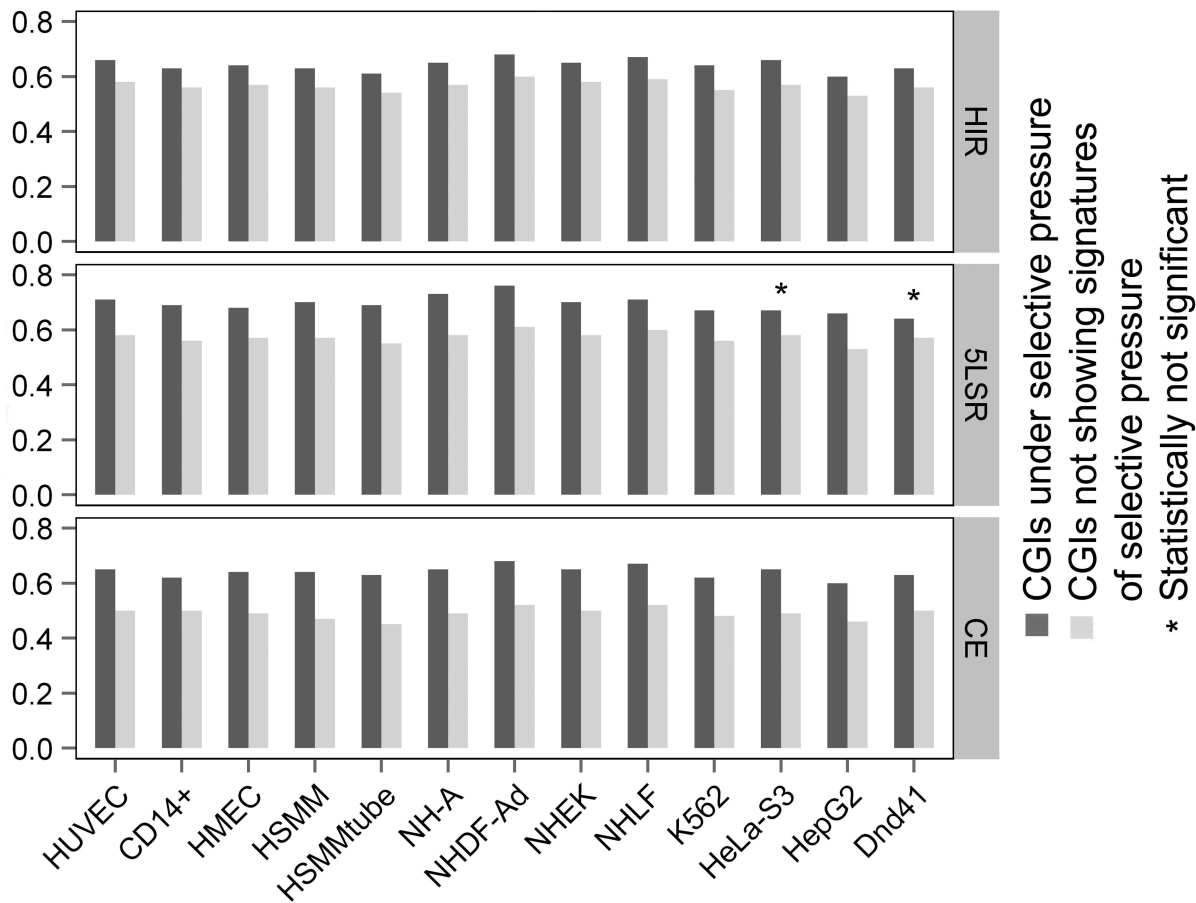

Supplement: Additional file 6 — Enrichment of H3K27ac modification in 5’ CpG islands under selective pressure. Same notation as Additional file 5. [file 1471-2148-13-145-S6.pdf]

# H3K36me3 enrichment in 5' CGIs

CGIs with peaks/CGIs

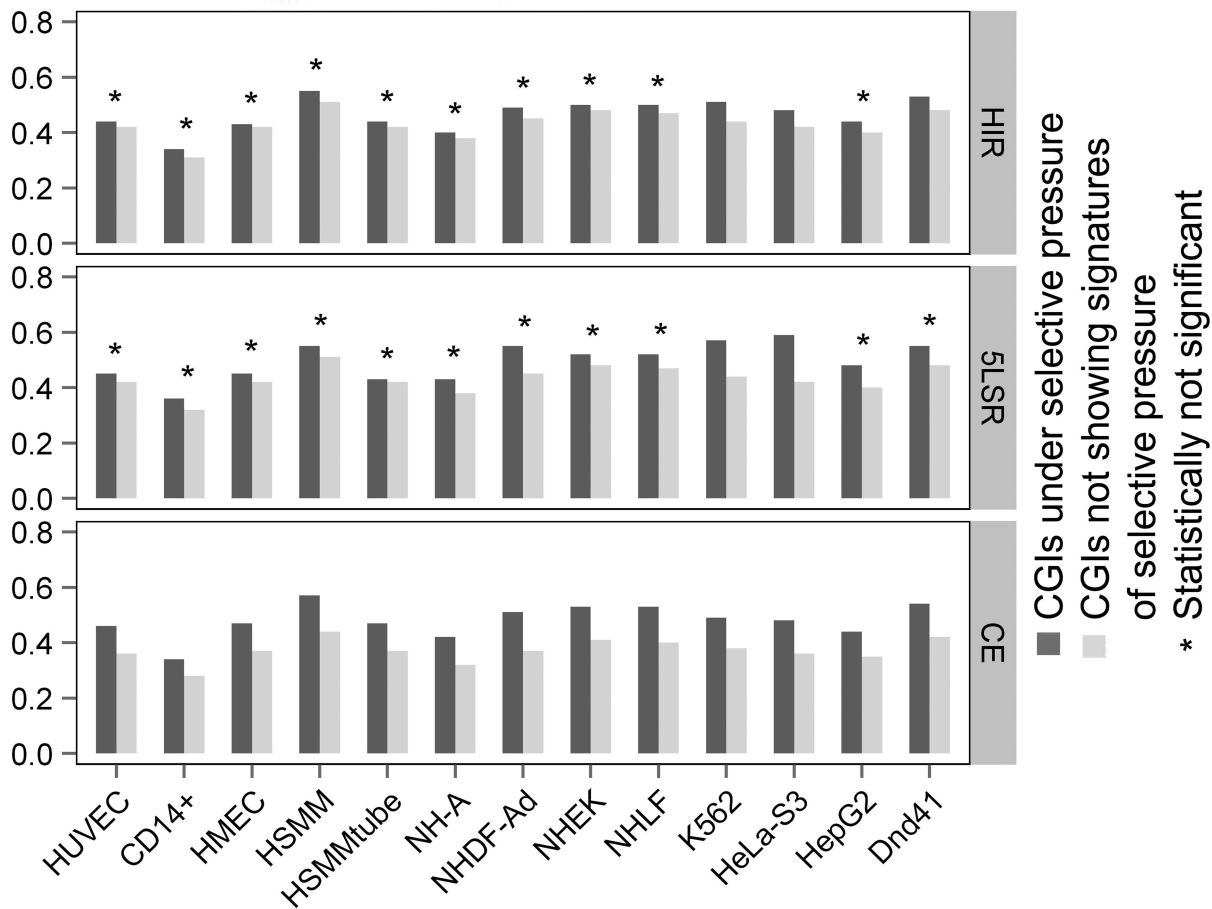

Supplement: Additional file 7 — Enrichment of H3K36me3 modification in 5’ CpG islands under selective pressure. Same notation as Additional file 5. [file 1471-2148-13-145-S7.pdf]

# H3K36me3 enrichment in intragenic CGIs

CGIs with peaks/CGIs

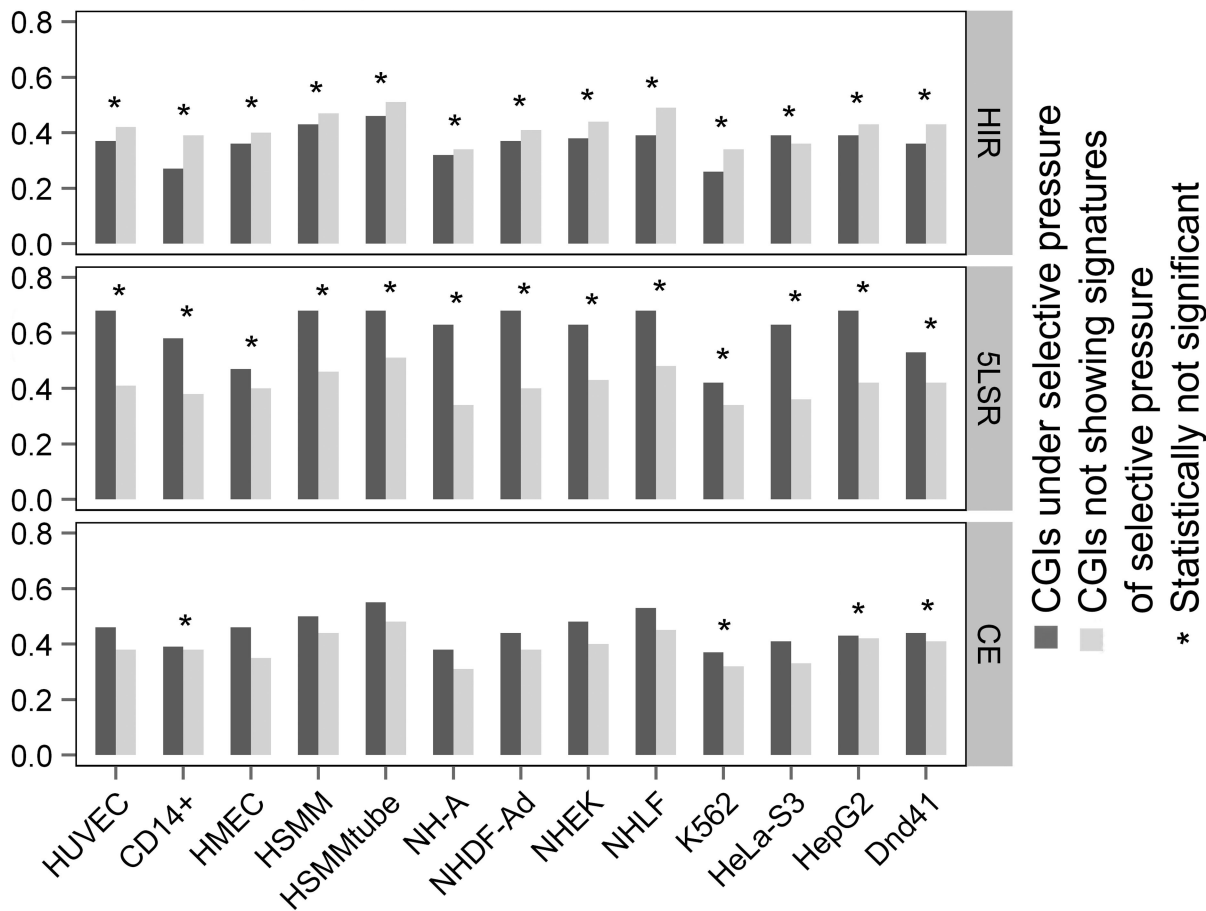

Supplement: Additional file 8 — Enrichment of H3K36me3 modification in intragenic CpG islands under selective pressure. Same notation as Additional file 5. [file 1471-2148-13-145-S8.pdf]

# H3K36me3 enrichment in 3' CGIs

CGIs with peaks/CGIs

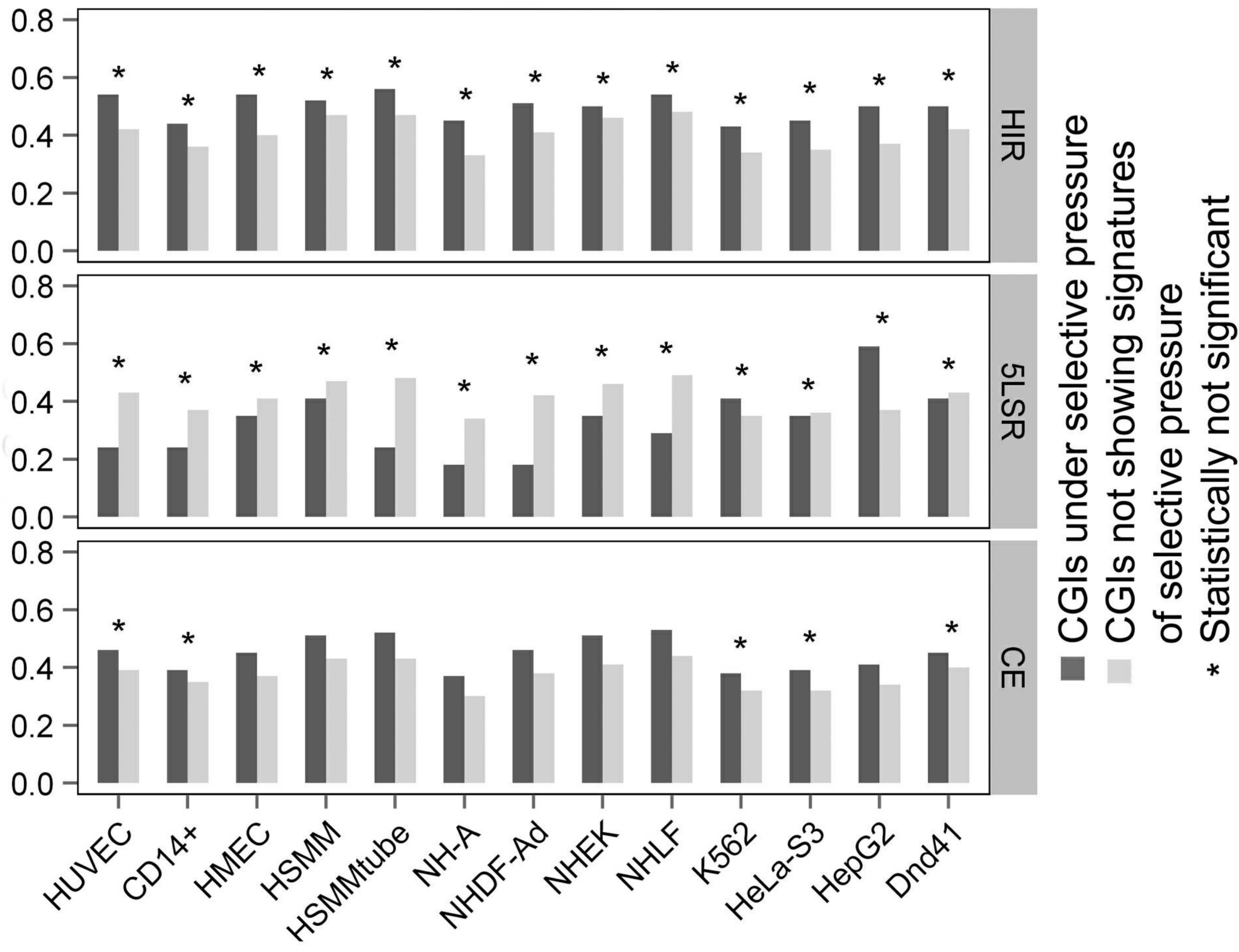

Supplement: Additional file 9 — Enrichment of H3K36me3 modification in 3’ CpG islands under selective pressure. Same notation as Additional file 5. [file 1471-2148-13-145-S9.pdf]

# H3K4me3 enrichment in intragenic CGIs

CGIs with peaks/CGIs

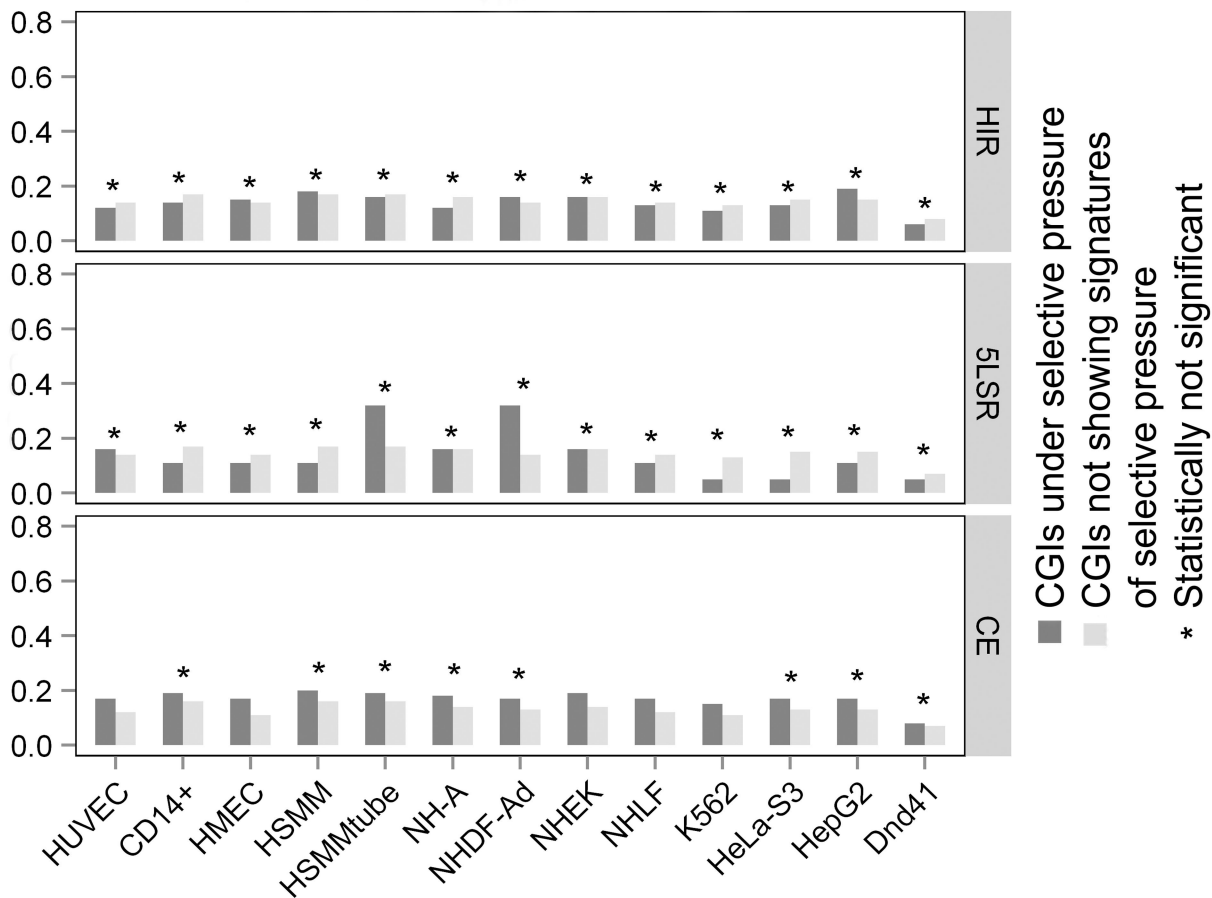

Supplement: Additional file 10 — Enrichment of H3K4me3 modification in intragenic CpG islands under selective pressure. Same notation as Additional file 5. [file 1471-2148-13-145-S10.pdf]

# H3K4me3 enrichment in 3' CGIs

CGIs with peaks/CGIs

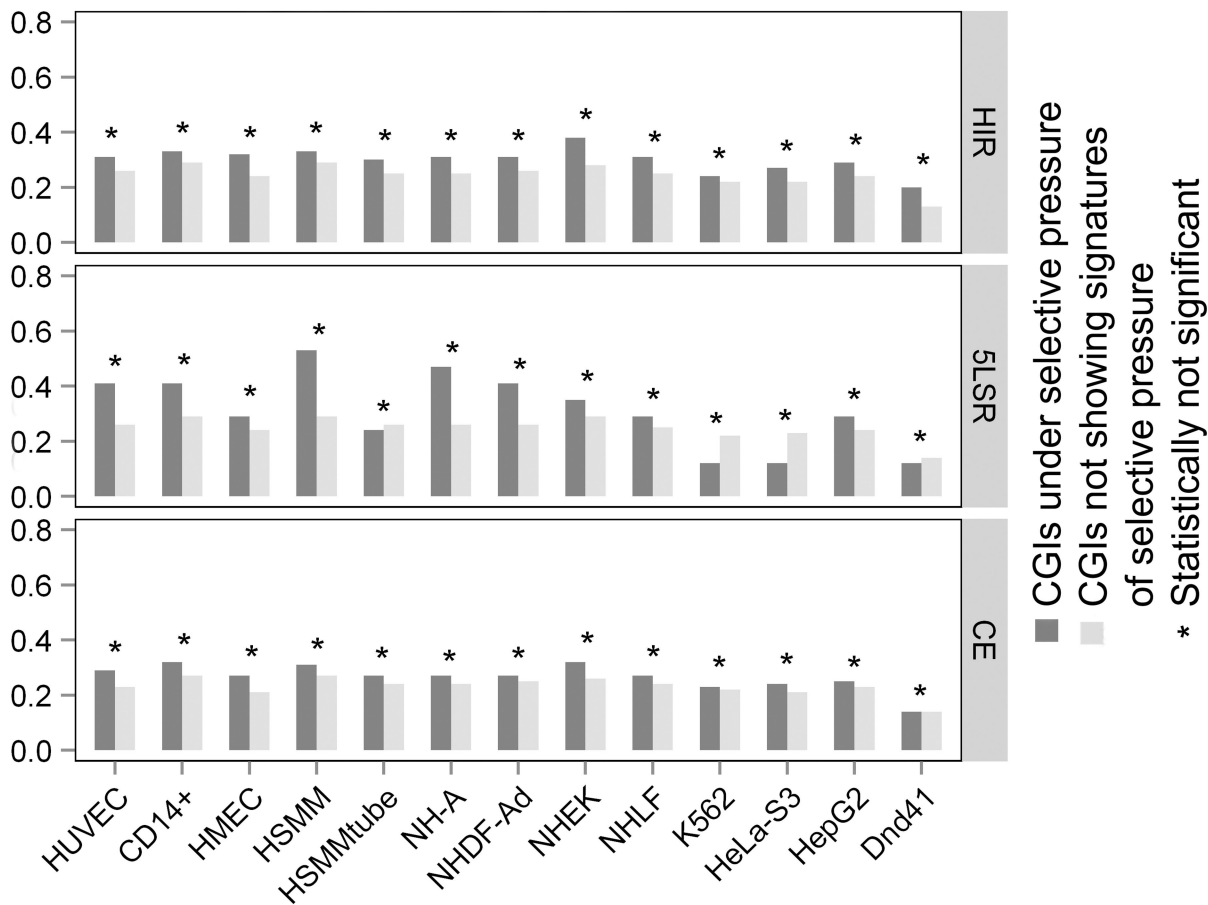

Supplement: Additional file 11 — Enrichment of H3K4me3 modification in 3’ CpG islands under selective pressure. Same notation as Additional file 5. [file 1471-2148-13-145-S11.pdf]

# H3K27ac enrichment in intragenic CGIs

CGIs with peaks/CGIs

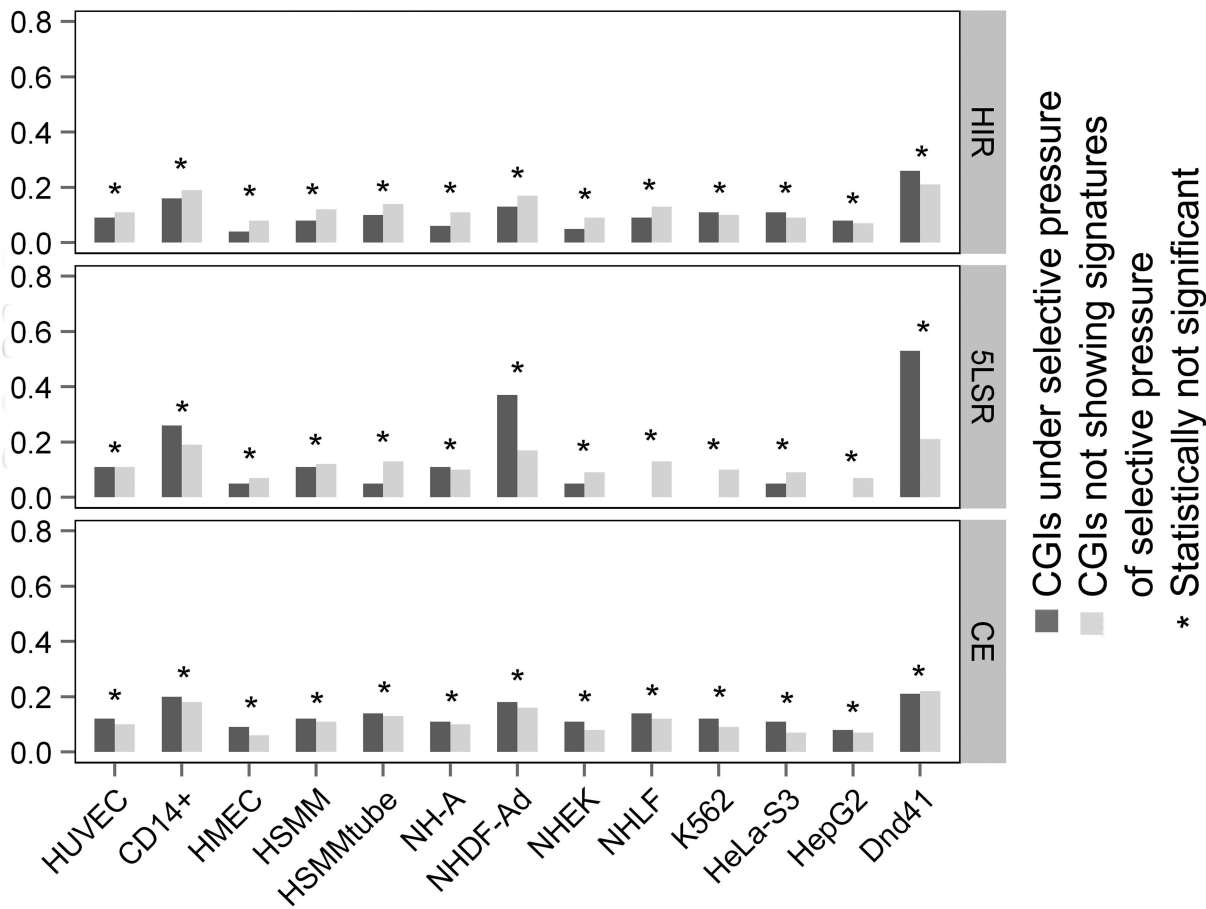

Supplement: Additional file 12 — Enrichment of H3K27ac modification in intragenic CpG islands under selective pressure. Same notation as Additional file 5. [file 1471-2148-13-145-S12.pdf]

# H3K27ac enrichment in 3' CGIs

CGIs with peaks/CGIs

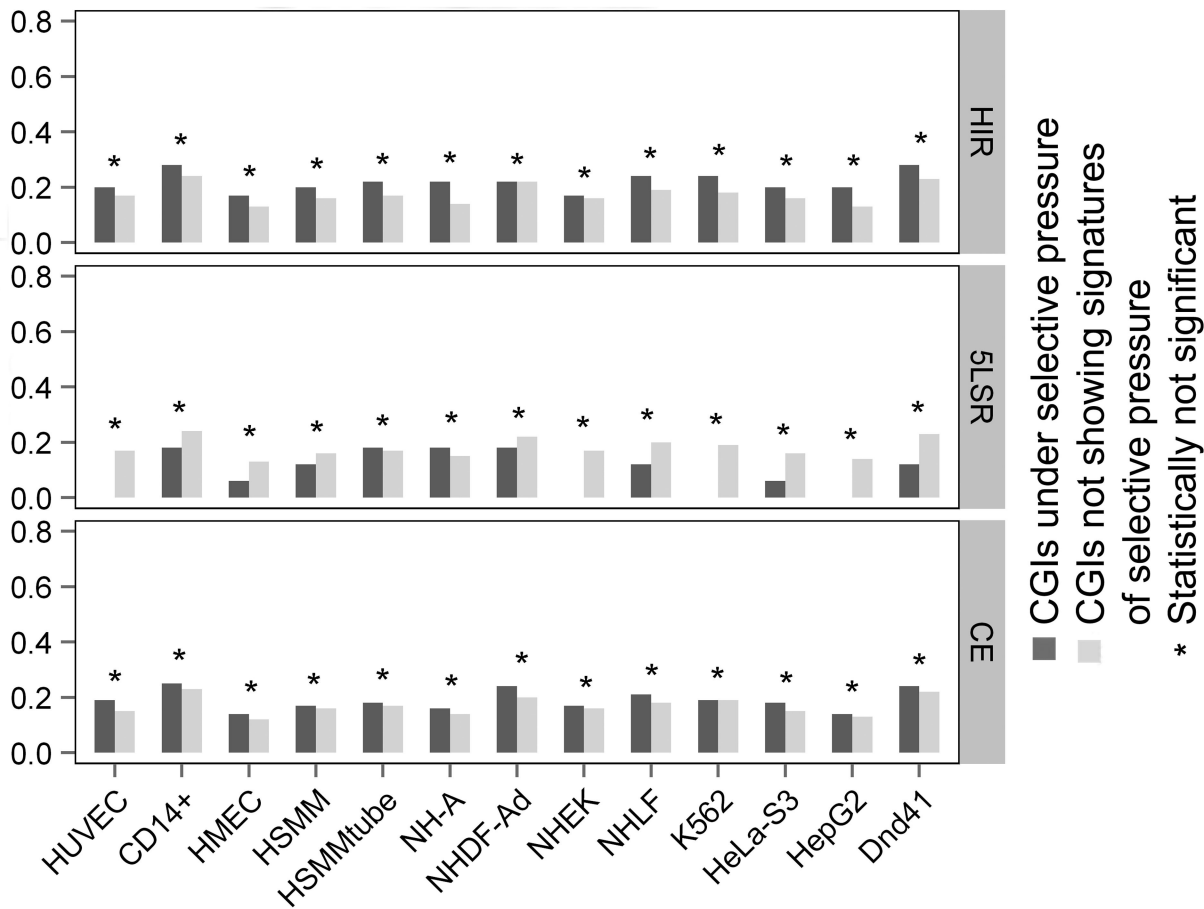

Supplement: Additional file 13 — Enrichment of H3K27ac modification in 3’ CpG islands under selective pressure. Same notation as Additional file 5. [file 1471-2148-13-145-S13.pdf]

# H3K4me3 enrichment in intergenic CGIs

CGIs with peaks/CGIs

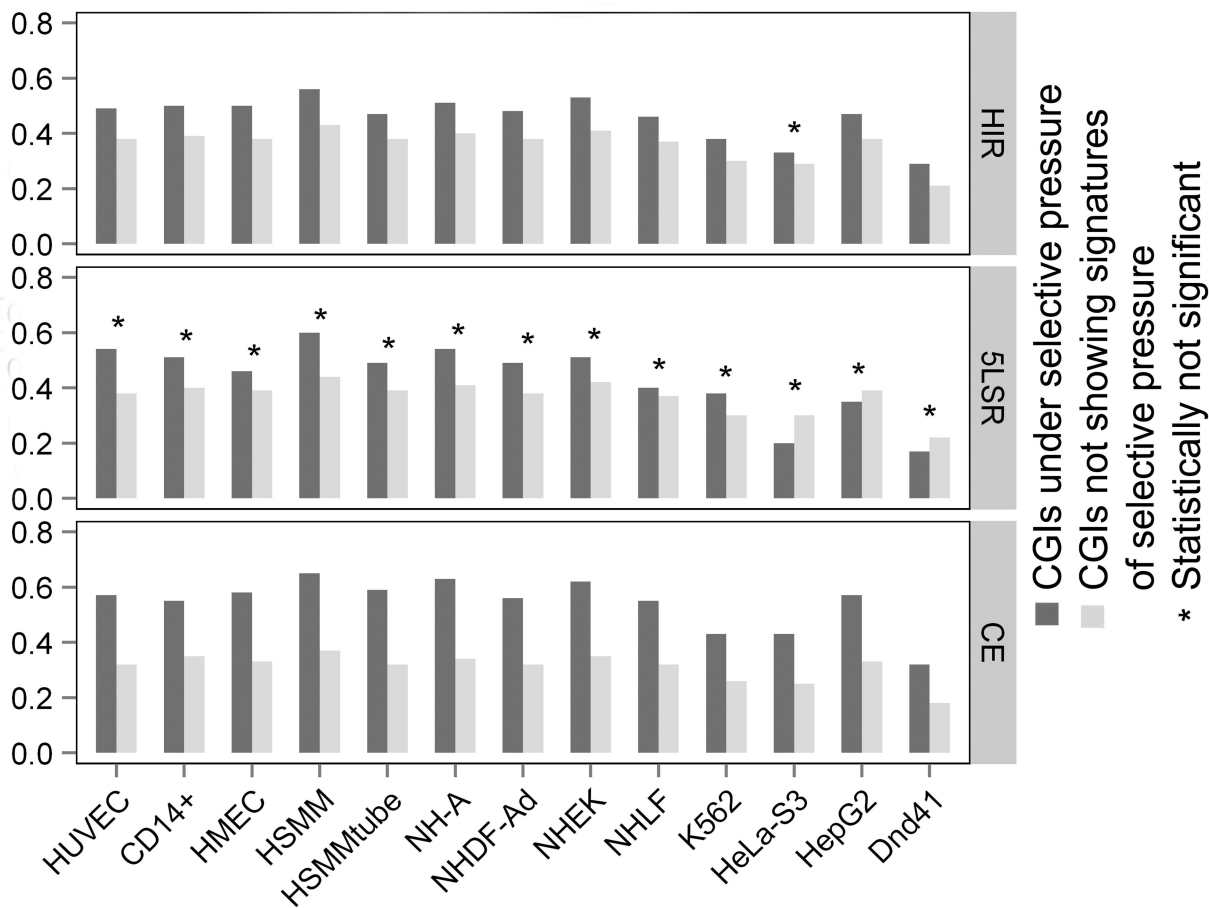

Supplement: Additional file 14 — Enrichment of H3K4me3 modification in intergenic CpG islands under selective pressure. Same notation as Additional file 5. [file 1471-2148-13-145-S14.pdf]

# H3K27ac enrichment in intergenic CGIs

CGIs with peaks/CGIs

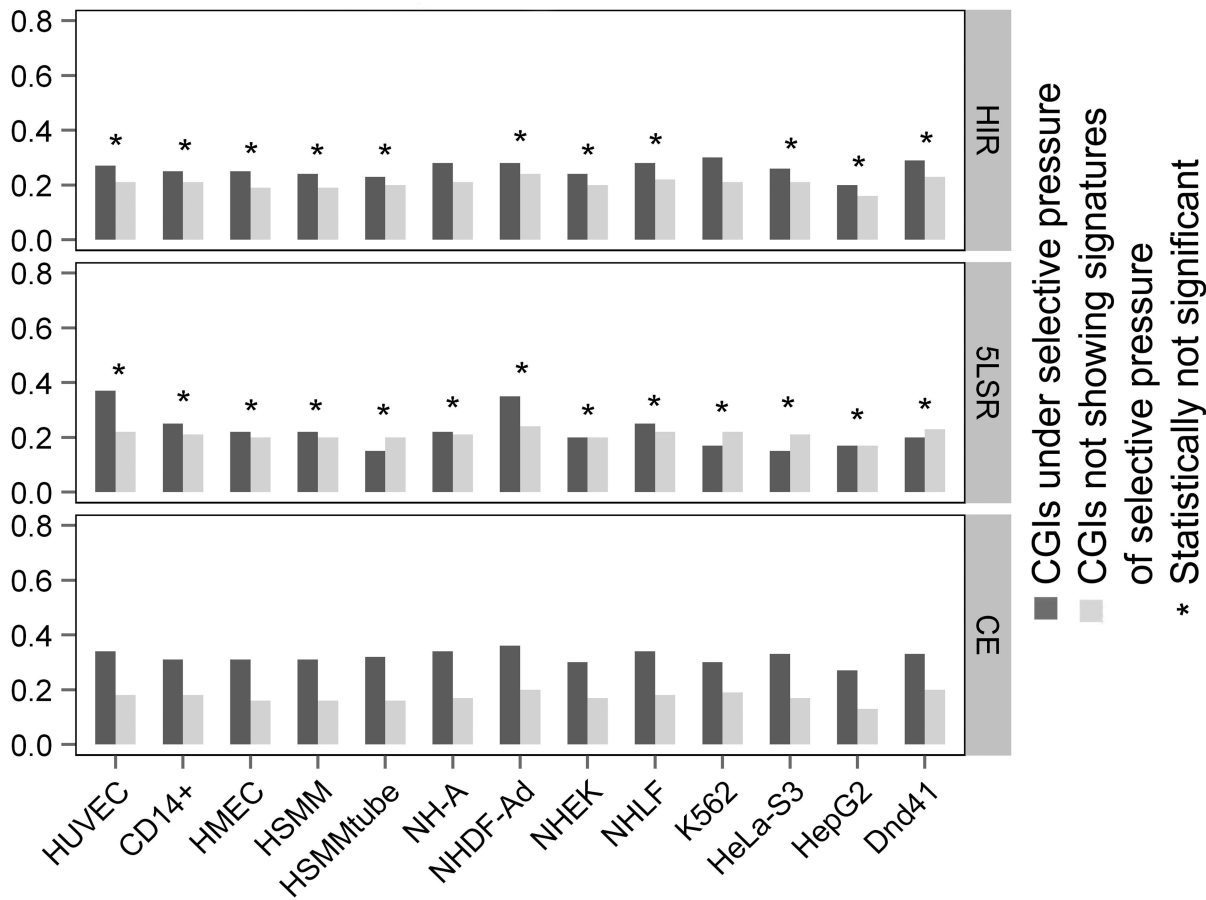

Supplement: Additional file 15 — Enrichment of H3K27ac modification in intergenic CpG islands under selective pressure. Same notation as Additional file 5. [file 1471-2148-13-145-S15.pdf]

# H3K36me3 enrichment in intergenic CGIs

CGIs with peaks/CGIs

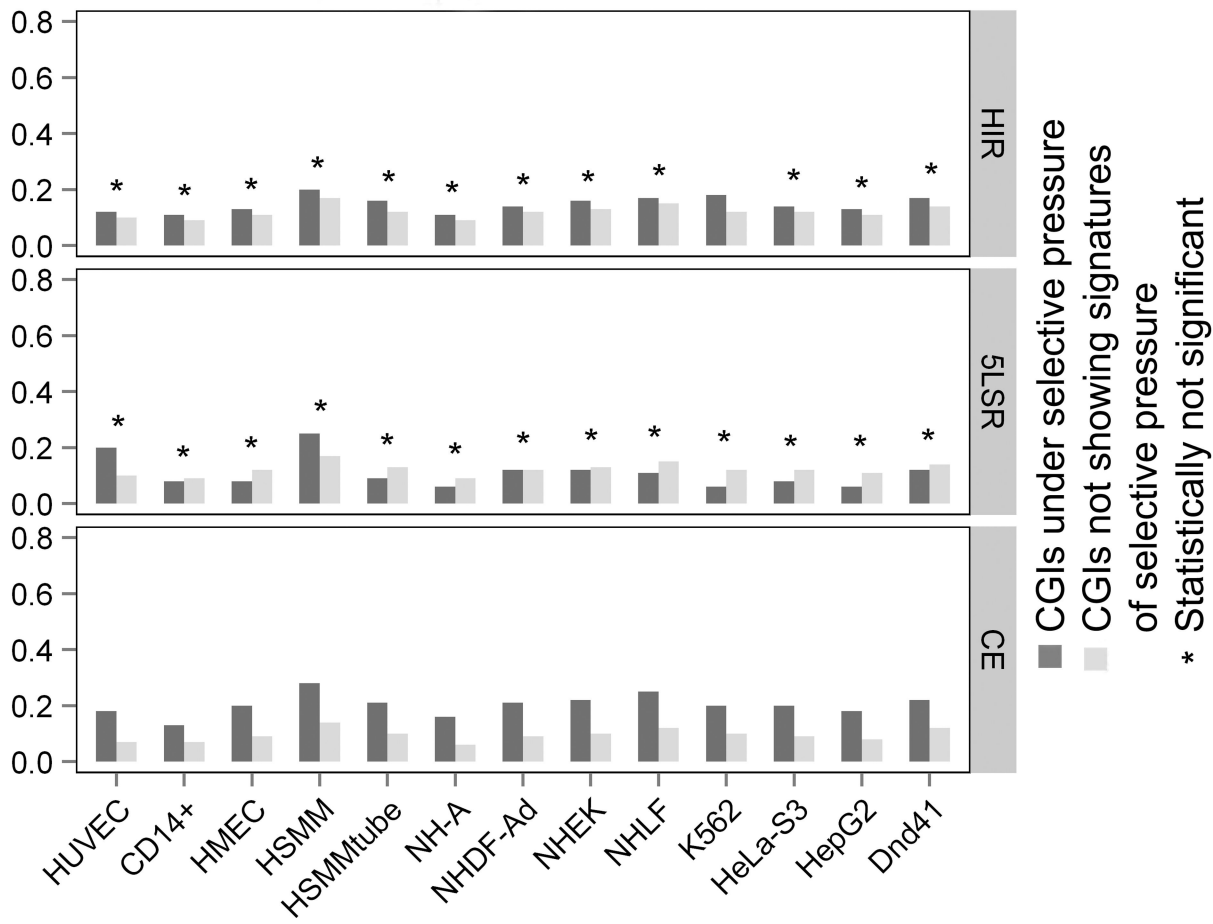

Supplement: Additional file 16 — Enrichment of H3K36me3 modification in intergenic CpG islands under selective pressure. Same notation as Additional file 5. [file 1471-2148-13-145-S16.pdf]

# H3K4me3 enrichment in hypo-deaminated CGIs

CGIs with peaks/CGIs

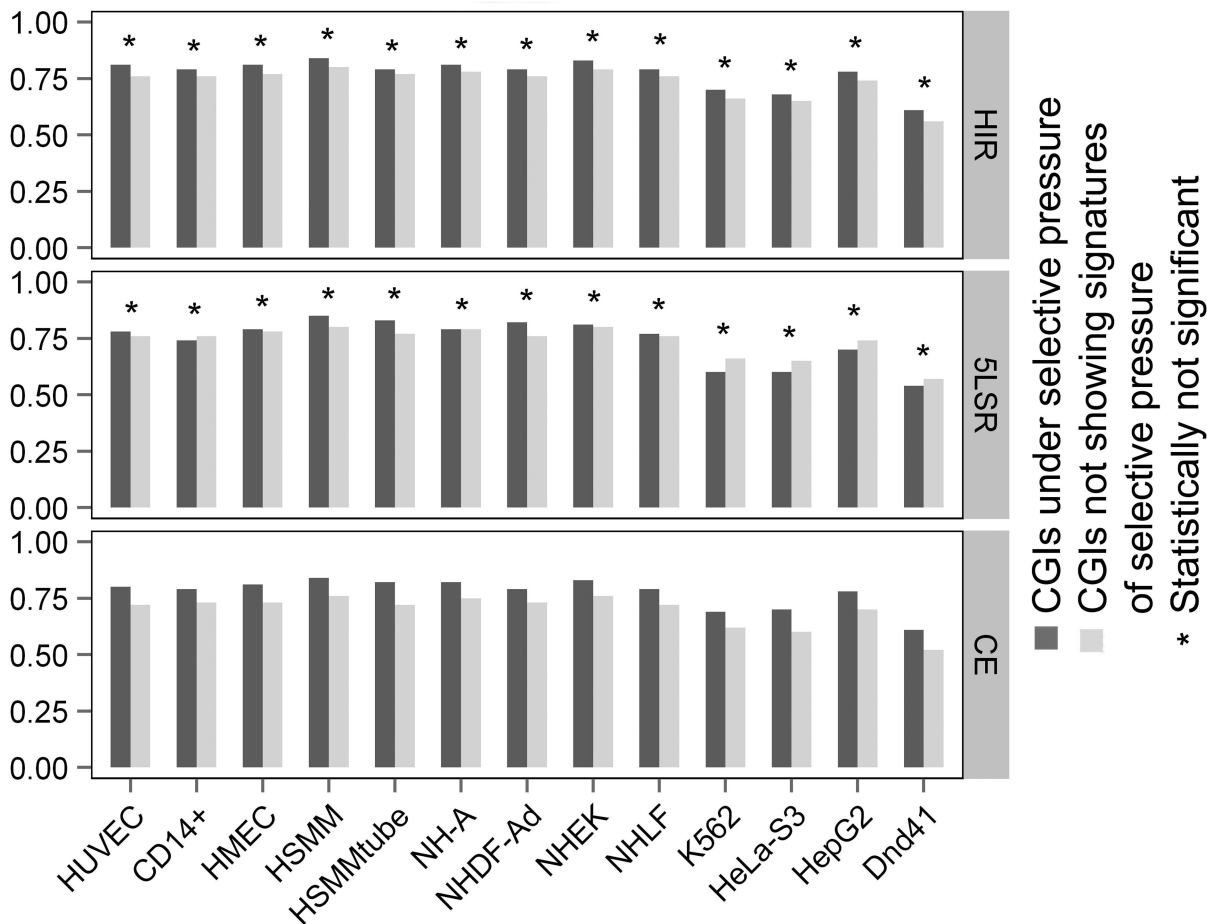

Supplement: Additional file 17 — Enrichment of H3K4me3 modification in hypo-deaminated CpG islands under selective pressure. Same notation as Additional file 5. [file 1471-2148-13-145-S17.pdf]

# H3K4me3 enrichment in BGC CGIs

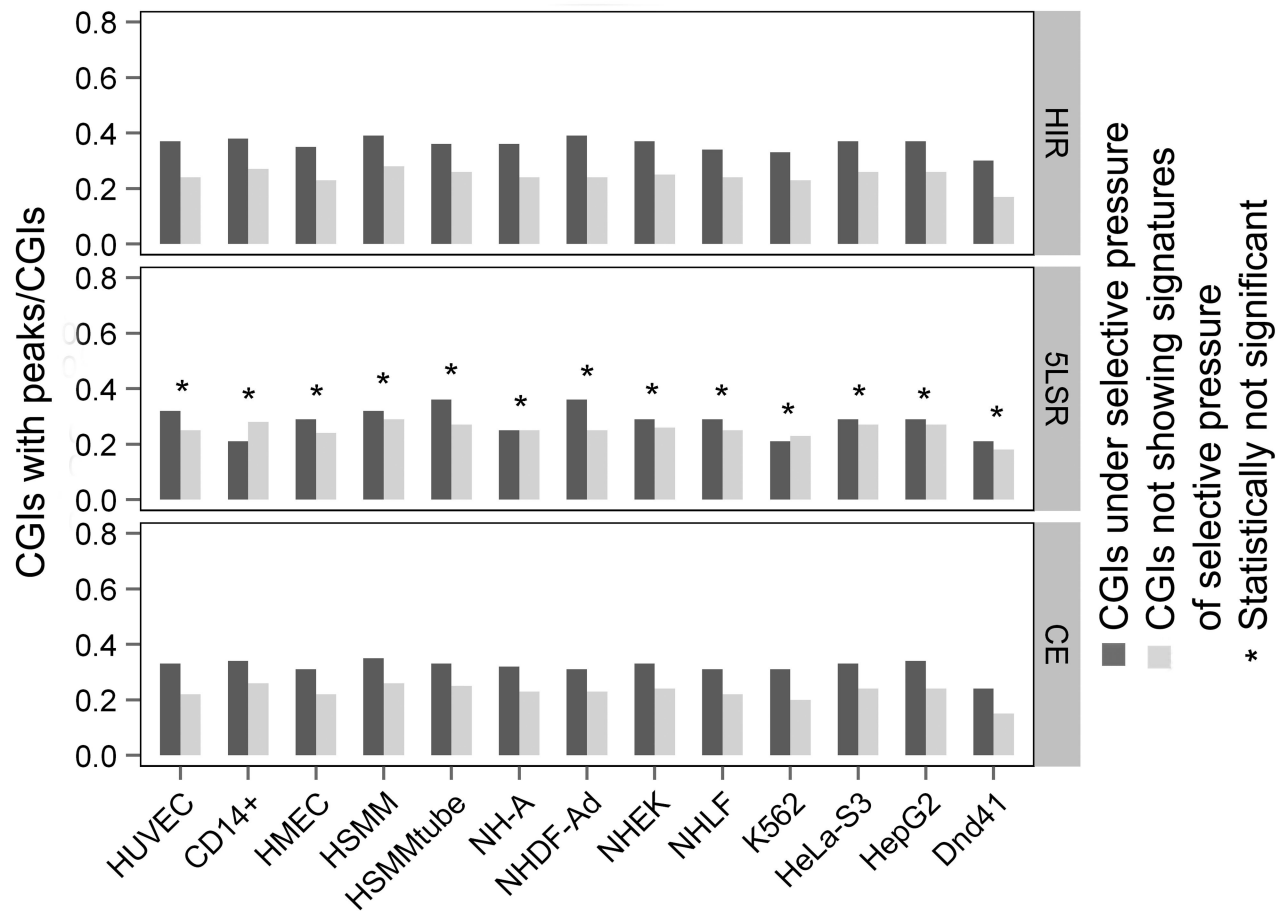

Supplement: Additional file 18 — Enrichment of H3K4me3 modification in BGC CpG islands under selective pressure. Same notation as Additional file 5. [file 1471-2148-13-145-S18.pdf]

# H3K27ac enrichment in BGC CGIs

CGIs with peaks/CGIs

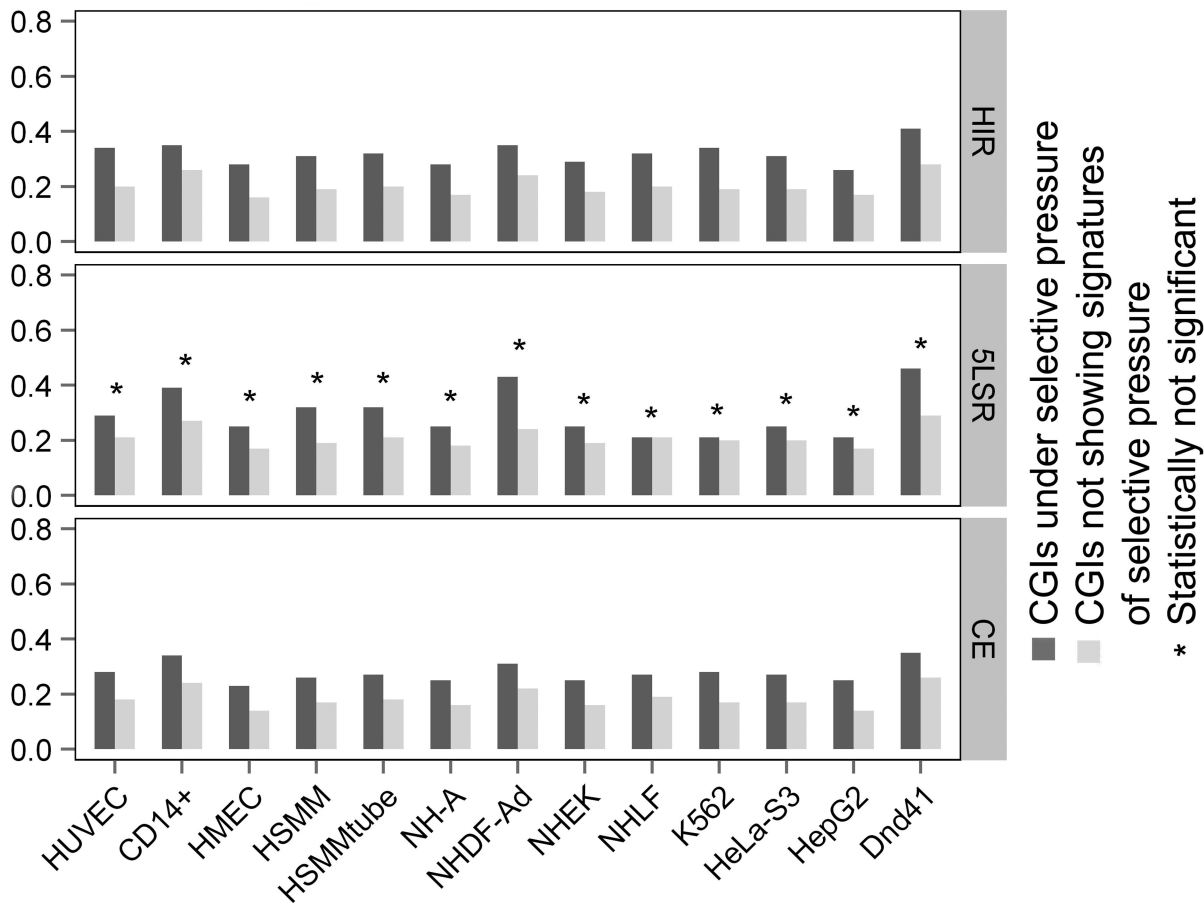

Supplement: Additional file 20 — Enrichment of H3K27ac modification in BGC CpG islands under selective pressure. Same notation as Additional file 5. [file 1471-2148-13-145-S20.pdf]

# H3K36me3 enrichment in hypo-deaminated CGIs

CGIs with peaks/CGIs

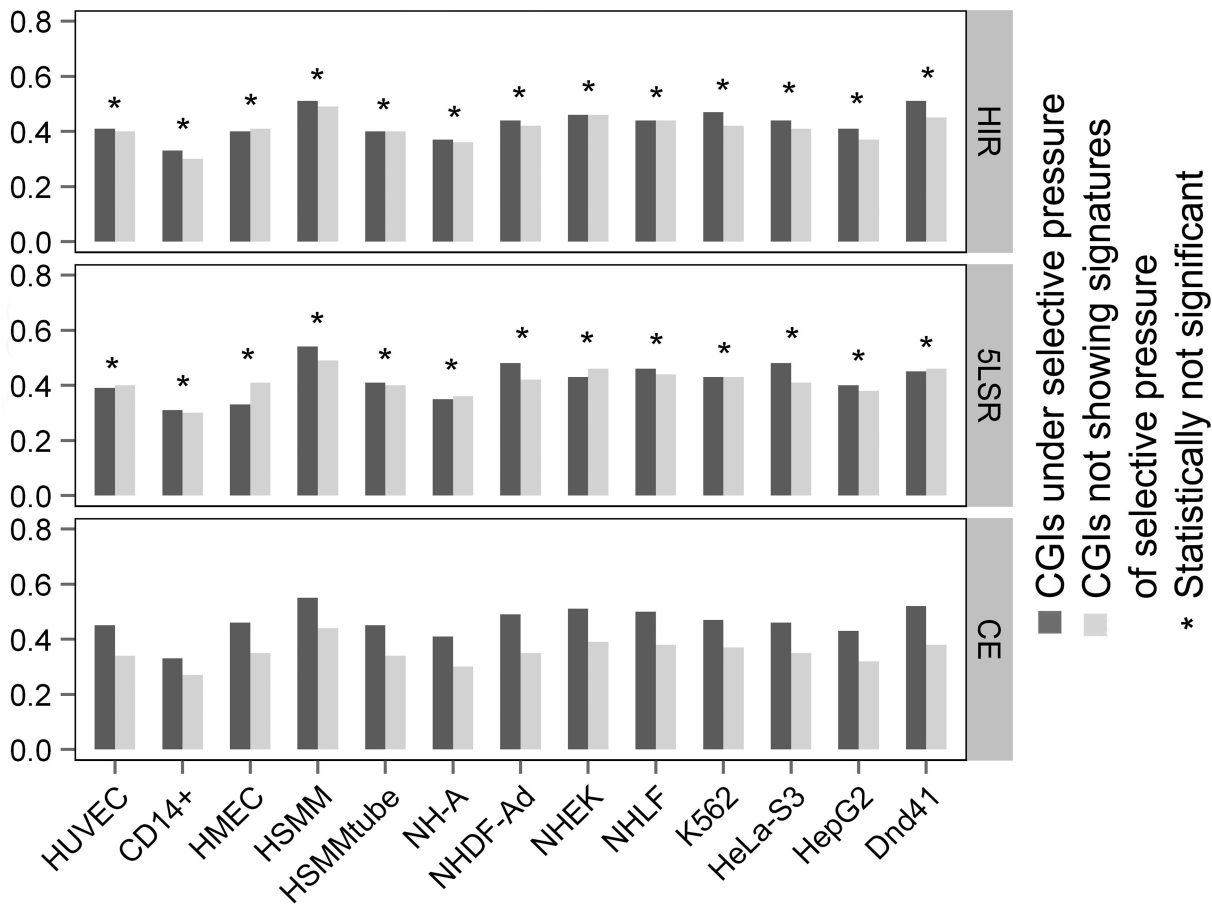

Supplement: Additional file 21 — Enrichment of H3K36me3 modification in hypo-deaminated CpG islands under selective pressure. Same notation as Additional file 5. [file 1471-2148-13-145-S21.pdf]

# H3K36me3 enrichment in BGC CGIs

CGIs with peaks/CGIs

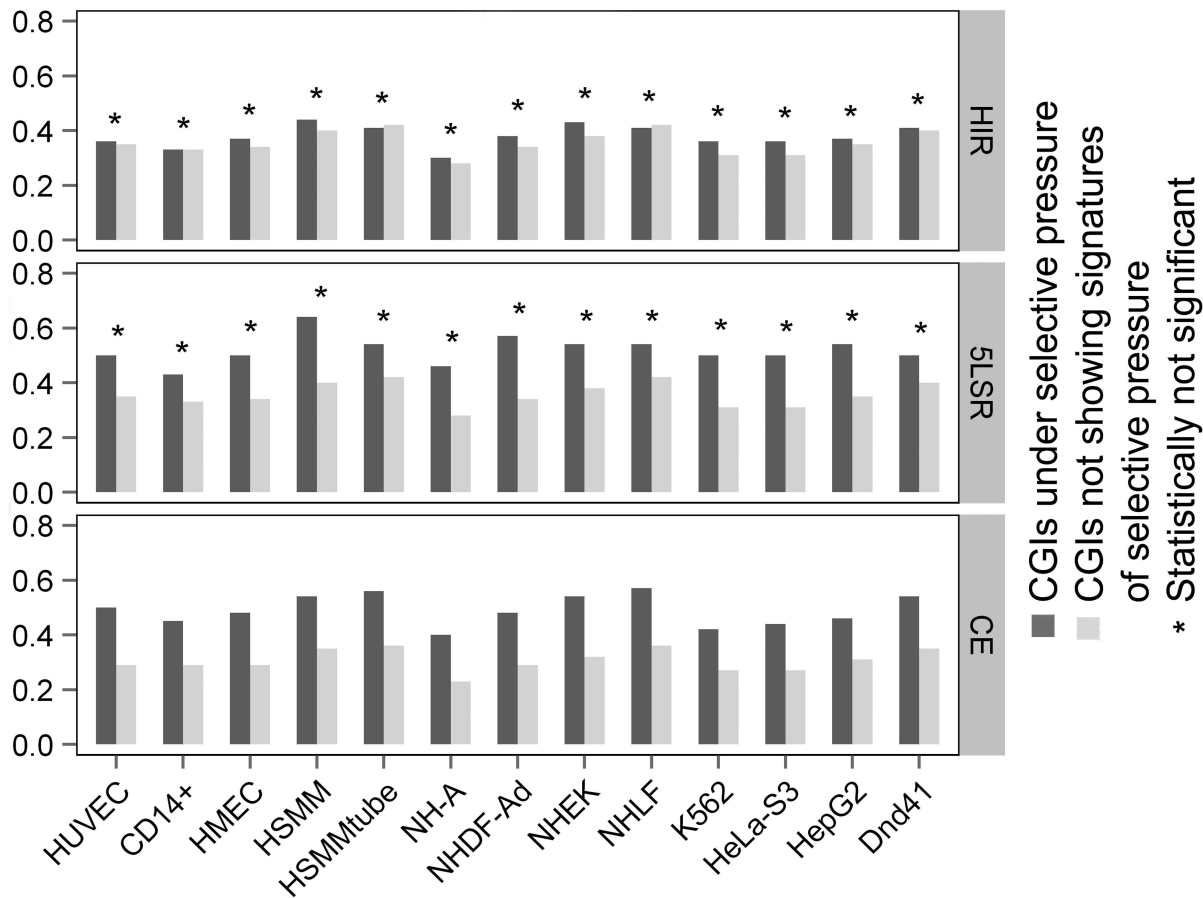

Supplement: Additional file 22 — Enrichment of H3K36me3 modification in BGC CpG islands under selective pressure. Same notation as Additional file 5. [file 1471-2148-13-145-S22.pdf]
