# Supplementary material for: CpG islands under selective pressure are enriched with H3K4me3, H3K27ac and H3K36me3 histone modifications
Source: BMC Evol Biol. 2013 Jul 10;13:145. doi: 10.1186/1471-2148-13-145 (PMC3711888; doi:10.1186/1471-2148-13-145)
Supplement: Additional file 5 — Enrichment of H3K4me3 modification in 5’ CpG islands under selective pressure. Black bars represent the fraction of CGIs containing histone modification marks within regions that show signatures of natural selection (HIRs, CEs and 5LSRs). Grey bars represent the fraction of CGIs containing histone modification marks within regions that do not show signatures of selective events. The X-axis indicates the analyzed cell lines. An asterisk (*) above a bar indicates a statistically non-significant difference. [file 1471-2148-13-145-S5.pdf]

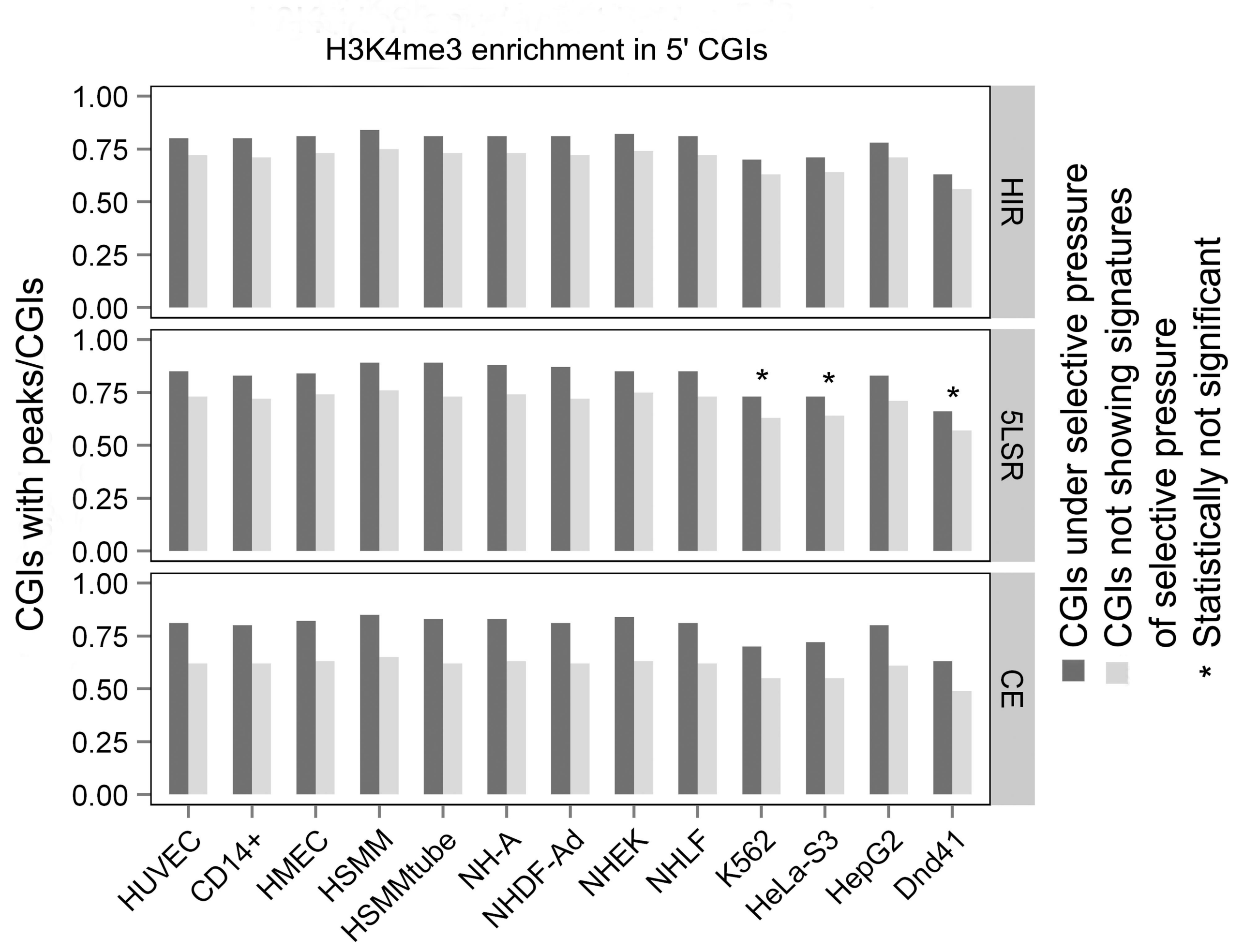
H3K4me3 enrichment in 5' CGIs
CGIs with peaks/CGIs
1.00
0.75
0.50
0.25
0.00
HIR
5LSR
CE
*
HUVEC
CD14+
HMEC
HSMM
HSMMtube
NH-A
NHDF-Ad
NHEK
NHLF
K562
HeLa-S3
HepG2
Dnd41
CGIs under selective pressure
CGIs not showing signatures of selective pressure
* Statistically not significant
