# Supplementary material for: CpG islands under selective pressure are enriched with H3K4me3, H3K27ac and H3K36me3 histone modifications
Source: BMC Evol Biol. 2013 Jul 10;13:145. doi: 10.1186/1471-2148-13-145 (PMC3711888; doi:10.1186/1471-2148-13-145)
Supplement: Additional file 19 — Enrichment of H3K27ac modification in hypo-deaminated CpG islands under selective pressure. Same notation as Additional file 5. [file 1471-2148-13-145-S19.pdf]

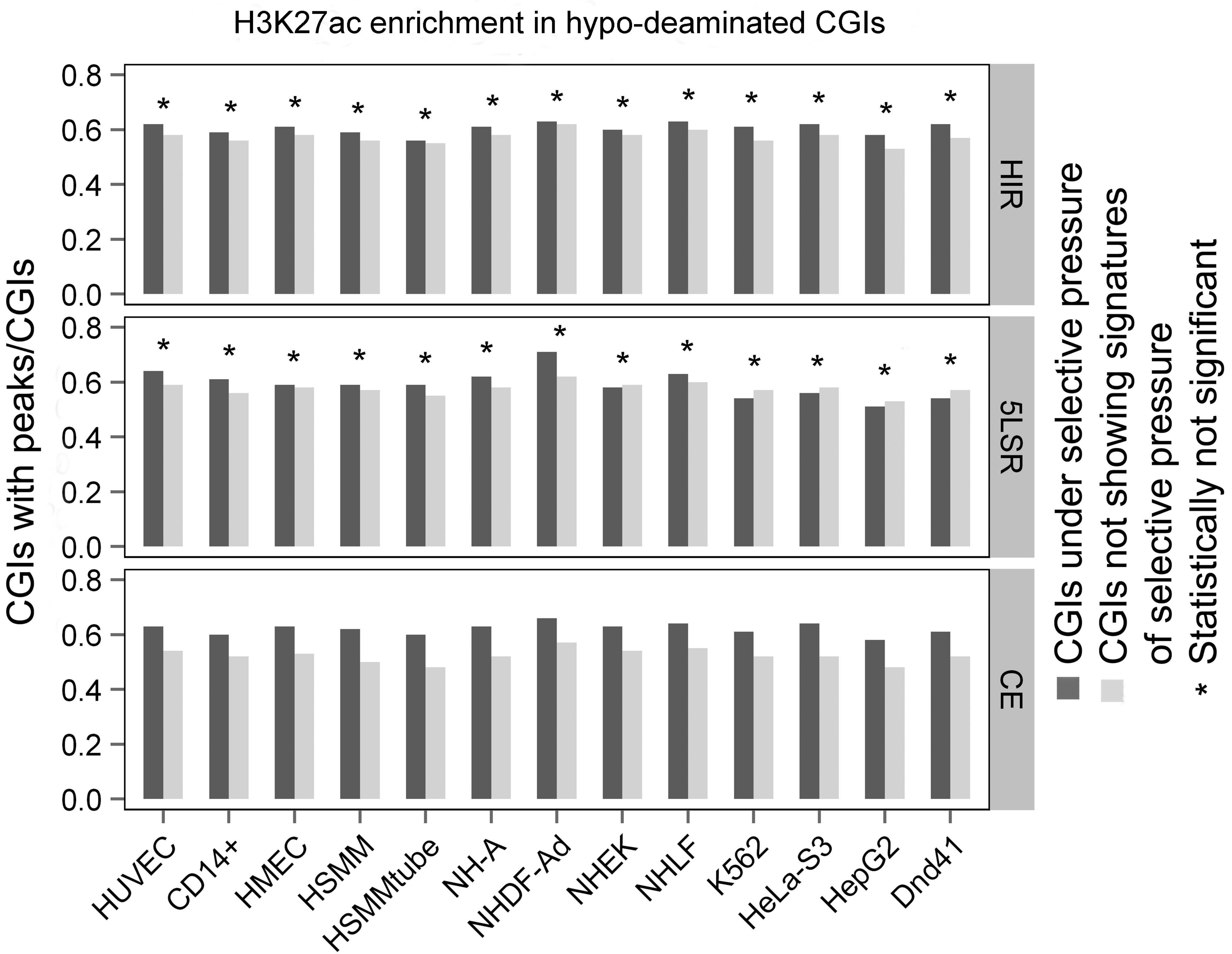

H3K27ac enrichment in hypo-deaminated CGIs
CGIs with peaks/CGIs
0.0
0.2
0.4
0.6
0.8
HIR
5LSR
CE
*
HUVEC
CD14+
HMEC
HSMM
HSMMtube
NH-A
NHDF-Ad
NHEK
NHLF
K562
HeLa-S3
HepG2
Dnd41
CGIs under selective pressure
CGIs not showing signatures of selective pressure
* Statistically not significant
